# HIF1α regulates glioma chemosensitivity through the transformation between differentiation and dedifferentiation in various oxygen levels

**DOI:** 10.1038/s41598-017-06086-2

**Published:** 2017-08-11

**Authors:** Pan Wang, Wenwu Wan, Shuanglong Xiong, Junwei Wang, Dewei Zou, Chuan Lan, Shuangjiang Yu, Bin Liao, Hua Feng, Nan Wu

**Affiliations:** 1 Department of Neurosurgery, Southwest Hospital, Third Military Medical University, Chongqing, 400038 China; 2 Department of Oncology, Cancer Hospital, Chongqing, 400030 China

## Abstract

Chemotherapy plays a significant role in glioma treatment; however, it has limited effectiveness in extending the life expectancies of glioma patients. Traditional studies have attributed this lack of efficacy to glioma stem cells (GSCs) and their high resistance to chemotherapy, and hypoxia worsens this issue. In contrast, hyperoxia effectively alleviates hypoxia in glioma and sensitizes glioma cells to chemotherapy. In a summary of traditional studies, the majority of researchers overlooked the influence of hypoxia on differentiated cells because they only focused on the maintenance of GSCs stemness, which thus resulted in chemoresistance. Because of this background, we hypothesized that GSCs may be induced through dedifferentiation under hypoxic conditions, and hypoxia maintains GSCs stemness, which thus leads to resistance to chemotherapy. In contrast, hyperoxia inhibits the dedifferentiation process and promotes GSCs differentiation, which increases the sensitization of glioma cells to chemotherapy. Hypoxia-inducible factor-1α (HIF1α) contributes substantially to the stemness maintenance of GSCs and resistance of glioma to chemotherapy; thus, we investigated whether HIF1α regulates the resistance or sensitization of glioma cells to chemotherapy in different oxygen levels. It highlights a novel viewpoint on glioma chemosensitivity from the transformation between dedifferentiation and differentiation in different oxygen levels.

## Introduction

Glioblastoma multiforme (GBM) is a highly malignant tumor in the brain and is characterized by rapid growth, resistance to conventional treatments and poor prognosis^1–3^. Temozolomide (TMZ) is a chemotherapeutic drug that has been widely used to treat GBM^1^. However, this strategy has limited effectiveness on extending the life expectancies of GBM patients^1, 2, 4, 5^. Traditional studies have attributed this finding to the presence of glioma stem cells (GSCs), which exhibit self-renewal without control and resistance to chemotherapy, including TMZ^1, 4, 6–9^. Researchers have shown that TMZ kills differentiated glioma cells and leaves GSCs intact, which thus results in chemoresistant GBM^6, 7, 10^. Another intrinsic factor with a substantial impact on glioma chemoresistance is the hypoxic microenvironment. Hypoxia promotes GSCs stemness, which leads to the high resistance to chemotherapy^11, 12^. However, an interesting phenomenon is that hypoxia increases the expression of CD133 for CD133^−^ glioma cells according to several studies^13, 14^. Therefore, two possibilities exist; one possibility is the enhanced CD133 originates from contaminated natural CD133^+^ cells, whereas the other possibility is that these GSCs originate from differentiated cancer cells through dedifferentiation under hypoxic conditions. However, hundreds of cells were cultured in these studies; thus, it remains unclear which scenario is correct.

Hyperoxia is an effective way to rectify glioma hypoxia and has been demonstrated to increase sensitivity to chemotherapy, including TMZ^15–17^. In 2012, Lu *et al*.^18^ reported that compared with TMZ or hyperbaric oxygen (HBO) alone, the combination of both treatments synergistically and significantly inhibited growth and induced apoptosis in U251 cells. These findings were in accordance with a recent study conducted by Dagistan *et al*.^19^, in which the combination of TMZ and HBO significantly decreased the levels of Ki67 in tumor tissue. However, the detailed mechanism requires further investigation. Based on the hypothesis that hypoxia induces the formation of GSCs through dedifferentiation and thus leads to resistance to TMZ, we hypothesize that hyperoxia inhibits dedifferentiation or promotes GSCs differentiation, which results in the sensitization of GBM cells to TMZ. Based on the significance of hypoxia-inducible factor-1a (HIF1α) in GSCs stemness maintenance^20, 21^, we determined the influence of HIF1α on the process of differentiation and dedifferentiation under different oxygen levels, which thus regulates the chemosensitivity of glioma cells.

## Results

### Glioma stem cells exhibited higher chemoresistance to TMZ

**Figure 1:**
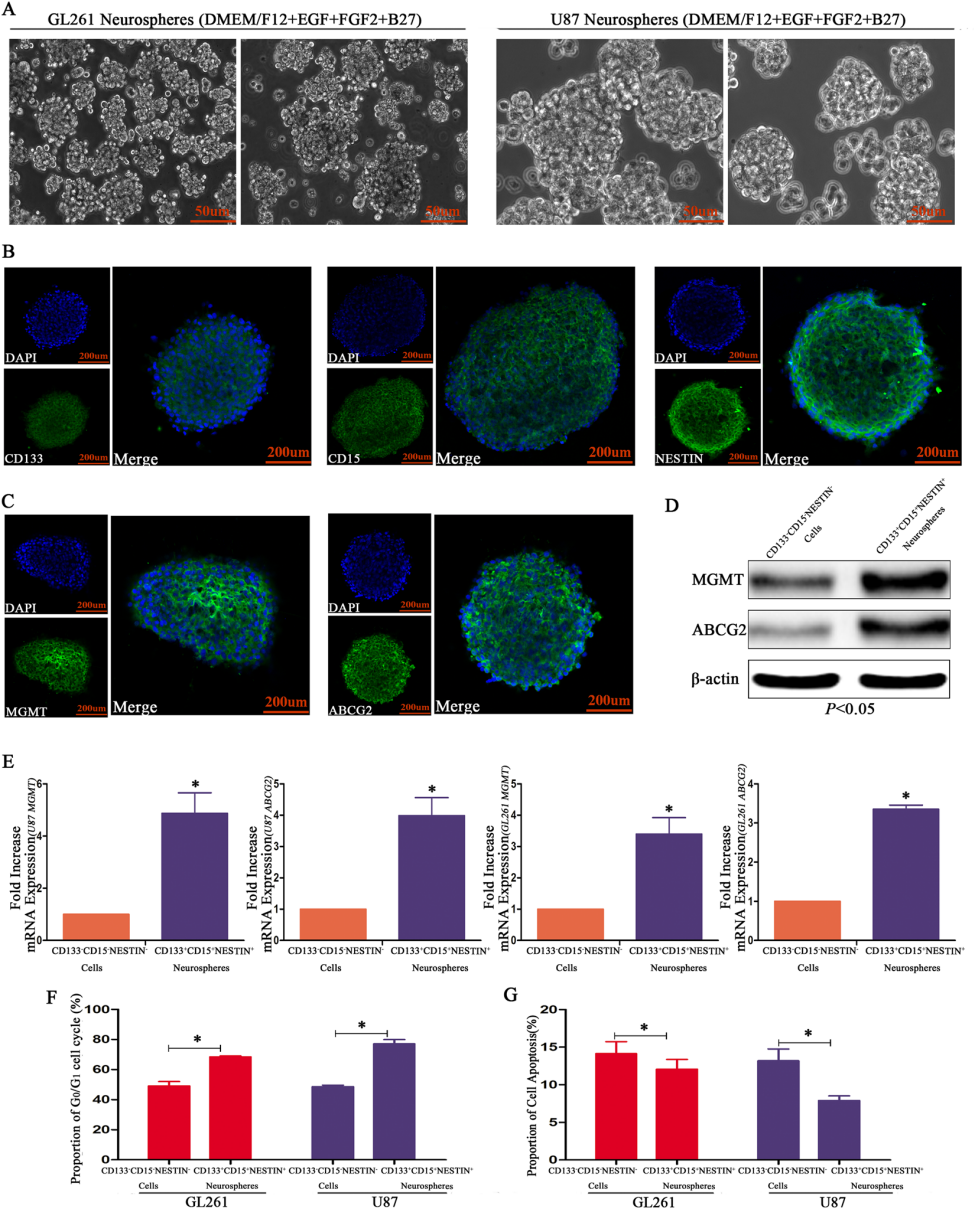
GSCs exhibited higher apoptosis rates than differentiated cells. (**A**) Sorted GL261 and U87 CD133^+^/CD15^+^/NESTIN^+^ GSCs were cultured in stem cell medium, and these cells grew with a sphere morphology in suspension. (**B**) U87 neurospheres highly expressed CD133, CD15 and NESTIN. (**C,D**) There was an increased expression of ABCG2 and MGMT in U87 neurospheres. (**E**) Three to five-fold higher expression levels of ABCG2 and MGMT were observed for GL261 and U87 CD133^+^/CD15^+^/NESTIN^+^ GSCs than CD133^−^/CD15^−^/NESTIN^−^ cells (^*^ *P* < 0.05, Paired-samples T Test). (**F**) GL261 and U87 CD133^+^/CD15^+^/NESTIN^+^ GSCs arrested the cell cycle in G_0_/G_1_ (^*^ *P* < 0.05, Paired-samples T Test). (**G**) Higher apoptosis rates were observed for GL261 and U87 CD133^−^/CD15^−^/NESTIN^−^ cells than for GSCs after TMZ (100 μM) treatment (^*^ *P* < 0.05, Paired-samples T Test).

CD133^+^CD15^+^NESTIN^+^ GSCs sorted from GL261 and U87 cells were cultured in stem cell medium (DMEM/F12 + EGF + FGF2 + B27), and the cells grew as a suspension with a sphere morphology (Fig. 1A). Immunofluorescence indicated these neurospheres highly expressed stem cell markers CD133, CD15 and NESTIN and the chemoresistance-related proteins ABCG2 and MGMT (Fig. 1B,C). Furthermore, western blot and RT-qPCR assays demonstrated an absolute increase in CD133, CD15, NESTIN, ABCG2 and MGMT expression in GSCs compared with CD133^−^CD15^−^NESTIN^−^ cells (Fig. 1D,E, Supplementary Figure S8A,B). We subsequently determined that the GSCs were arrested in G_0_/G_1_ (Fig. 1F), and fewer of these cells underwent apoptosis after TMZ (100 μM) exposure compared with CD133^−^CD15^−^NESTIN^−^ cells exposed to the same treatments (Fig. 1G).

### Chemoresistance-related protein detection in different oxygen levels

**Figure 2:**
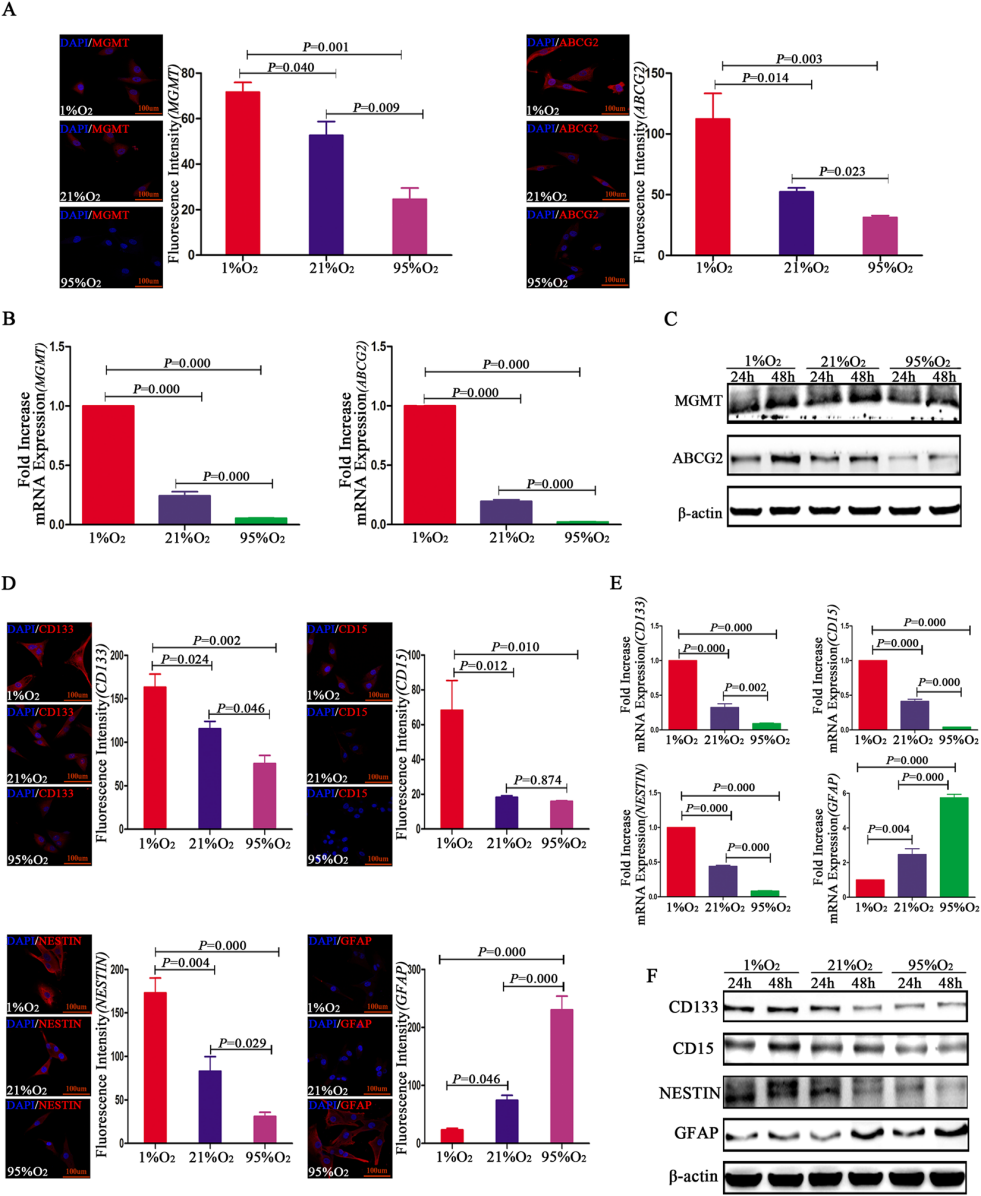
Protein detection for CD133^−^/CD15^−^/NESTIN^−^ glioma cells under different oxygen levels. (**A**) Higher MGMT and ABCG2 levels were detected for GL261 CD133^−^CD15^−^NESTIN^−^ cells under 1%O_2_ conditions for 24 h than 21%O_2_ or 95%O_2_ via immunofluorescence staining. Cells demonstrated the lowest expression in 95%O_2_ (*P* < 0.05, One-way ANOVA). (**B**) A 5-fold increase of MGMT in 1%O_2_ was identified for GL261 CD133^−^CD15^−^NESTIN^−^ cells via RT-qPCR compared with normoxia, and MGMT was fewer in 95%O_2_. Similar results were obtained for ABCG2 in different oxygen levels (*P* < 0.05, One-way ANOVA). (**C**) There were significant differences in MGMT and ABCG2 for GL261 CD133^−^CD15^−^NESTIN^−^ cells under different oxygen levels in western blot; the highest expression was identified in 1%O_2_, and the lowest expression was demonstrated in 95%O_2_ (*P* < 0.05, One-way ANOVA). Both MGMT and ABCG2 increased levels in a time dependent manner from hypoxia for 24 to 48 h. However, under hyperoxia, MGMT expression remained stable; for ABCG2, there was lower expression after hyperoxia treatment (*P* < 0.05, One-way ANOVA). (**D**) Compared with 21%O_2_ or 95%O_2_, increased CD133, CD15 and NESTIN levels were identified for GL261 CD133^−^CD15^−^NESTIN^−^ cells in 1%O_2_ via immunofluorescence staining. Cells demonstrated the lowest expression after exposure in 95%O_2_. In contrast, GFAP was highly expressed after hyperoxia exposure for 24 h, which exhibited 3-fold increases compared with normoxia and 12-fold compared with hypoxia. There was almost no GFAP expression in the 1%O_2_ or 21%O_2_ microenvironments (*P* < 0.05, One-way ANOVA). (**E**) RT-qPCR indicated the CD133, CD15 and NESTIN levels significantly increased in 1%O_2_ for 12 h compared with 21%O_2_ and 95%O_2_ for GL261 CD133^−^CD15^−^NESTIN^−^ cells. However, the GFAP levels increased in an oxygen concentration-dependent manner, and GFAP was 3-fold higher under hyperoxia than normoxia and 6-fold higher than hypoxia. A significant difference was also identified between the 1%O_2_ and 21%O_2_ microenvironments (*P* < 0.05, One-way ANOVA). (**F**) GL261 CD133^−^CD15^−^NESTIN^−^ cells presented the highest expression of CD133, CD15 and NESTIN after 1%O_2_ exposure for 24 or 48 h. Furthermore, CD133, CD15 and NESTIN increased levels in a time-dependent manner from hypoxia for 24 to 48 h; however, under 21%O_2_ conditions, the expression levels significantly decreased for CD133 and NESTIN in a time-dependent manner. Under 95%O_2_, CD133, CD15 and NESTIN were the lowest expression. In contrast, GFAP was highly expressed in 21%O_2_ and 95%O_2_ with lower detection under 1%O_2_ conditions. Furthermore, the GFAP levels increased in a time-dependent manner from normoxia or hyperoxia for 24 to 48 h (*P* < 0.05, One-way ANOVA).

Immunofluorescence indicated that compared with 21%O_2_ or 95%O_2_, MGMT and ABCG2 were more highly expressed in GL261 CD133^−^CD15^−^NESTIN^−^ cells exposed to 1% O_2_ (Fig. 2A). RT-qPCR indicated that after hypoxia exposure for 12 h, the expression of MGMT in GL261 CD133^−^CD15^−^NESTIN^−^ cells was approximately 5-fold higher than that in those exposed to normoxia, and the mRNA levels was the lowest in cells exposed to 95%O_2_ (Fig. 2B). A similar result was obtained for ABCG2 in cells exposed to different oxygen levels (Fig. 2B). Western blot assays subsequently indicated GL261 CD133^−^CD15^−^NESTIN^−^ cells presented higher expression levels of MGMT and ABCG2 after hypoxia exposure for 24 or 48 h compared with those exposed to normoxia and hyperoxia (Fig. 2C; Supplementary Figure S1A). Furthermore, the expression of both MGMT and ABCG2 increased in cells exposed to hypoxia in a time-dependent manner 24 to 48 h. However, under hyperoxia, MGMT expression remained stable, and ABCG2 was lower expression (Fig. 2C; Supplementary Figure S1B).

### GSCs and differentiated-related protein detection under different oxygen levels

Immunofluorescence indicated that GL261 CD133^−^CD15^−^NESTIN^−^ cells had an increased expression of CD133, CD15 and NESTIN after hypoxia exposure for 24 h compared with those exposed to normoxia and hyperoxia. Moreover, compared with hyperoxia, CD133 and NESTIN were significantly increased at least two-fold after normoxia exposure for 24 h. However, GFAP, as a differentiated marker of glioma cells, was highly expressed in cells exposed to hyperoxia and exhibited a 3-fold increase compared with those exposed to normoxia and 12-fold compared with hypoxia. There was nearly few GFAP expressed in cells exposed to 1%O_2_ or 21%O_2_ conditions (Fig. 2D). Moreover, RT-qPCR indicated that the levels of CD133, CD15 and NESTIN increased in GL261 CD133^−^CD15^−^NESTIN^−^ cells exposed to 1% O_2_ compared with those exposed to 21%O_2_ and 95%O_2_. CD133, CD15 and NESTIN were not expressed in hyperoxia conditions. However, the GFAP levels increased in an oxygen concentration-dependent manner, as GFAP expression was 3-fold higher under hyperoxia compared with normoxia and 6-fold higher compared with hypoxia (Fig. 2E). Western blot assays subsequently demonstrated that GL261 CD133^−^CD15^−^NESTIN^−^ cells presented higher expression levels of CD133, CD15 and NESTIN after 1%O_2_ exposure for 24 or 48 h than cells exposed to normoxia or hyperoxia. Moreover, the CD133, CD15 and NESTIN levels increased in a time-dependent manner under 1%O_2_ conditions, whereas under 21%O_2_, the expression significantly decreased for CD133 and NESTIN in a time-dependent manner from 24 to 48 h. Under hyperoxia, there was the lowest expression of CD133, CD15 or NESTIN. In contrast, GFAP was highly expressed under 21%O_2_ and 95%O_2_ conditions, with lower detection in 1%O_2_. Furthermore, GFAP increased in a time-dependent manner from normoxia or hyperoxia 24 to 48 h (Fig. 2F; Supplementary Figure S1C,D).

### Growth characteristics for single CD133^−^CD15^−^NESTIN^−^ glioma cell under different oxygen levels

**Figure 3:**
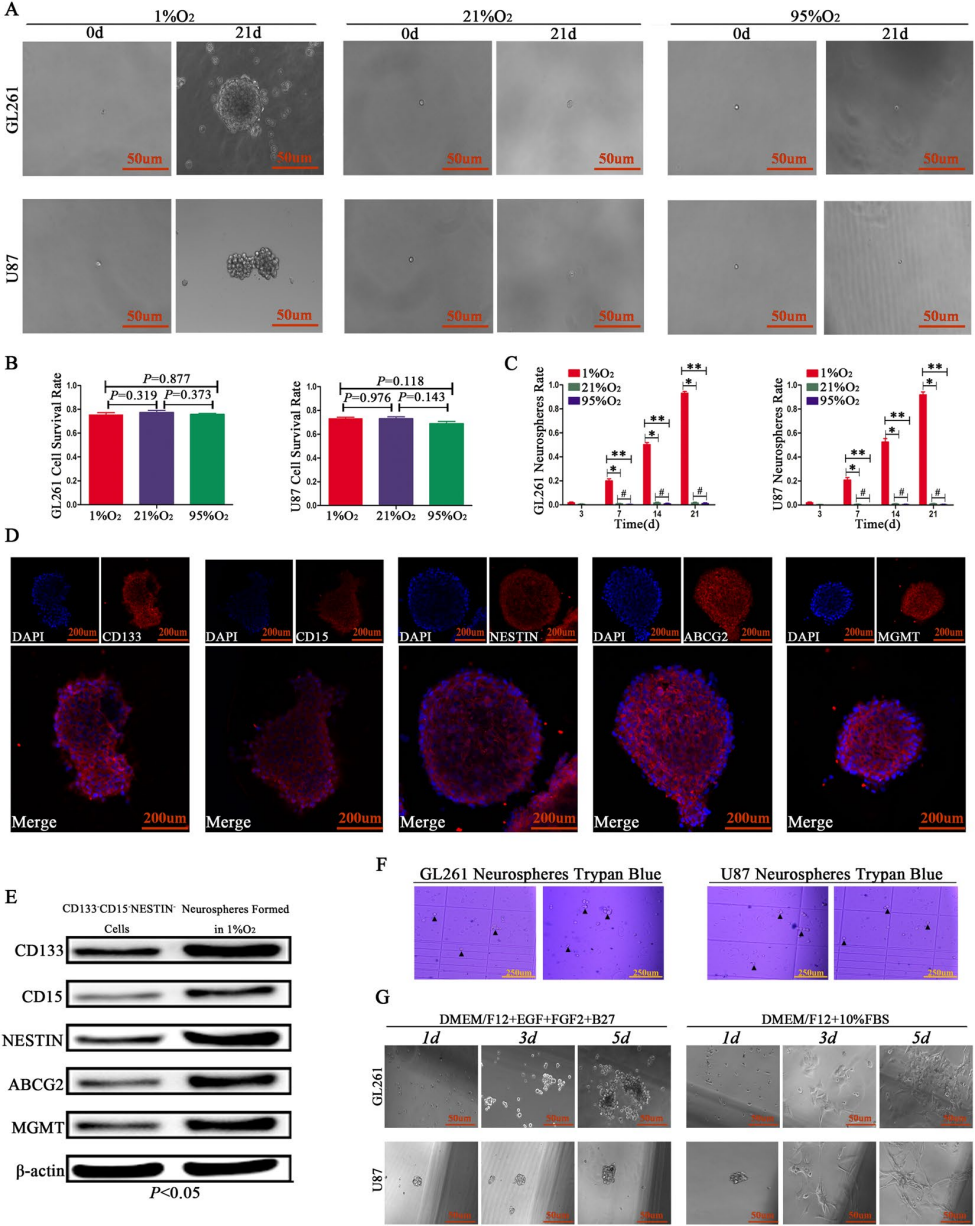
Neurospheres formed in 1%O_2_ presented with features of GSCs. (**A–C**) Single GL261 and U87 CD133^−^CD15^−^NESTIN^−^ cell was seeded in the wells of 96-well plates and cultured in 1%O_2_, 21%O_2_ and 95%O_2_. Approximately 75% of cells survived after exposure to different oxygen levels for 3 days without a significant difference (d3 viable cells/d0 seeding cells). The neurospheres began to form after 1%O_2_ exposure for 3 d, and approximately 20% of cells (d7 spheres/d3 surviving cells) from GL261 and U87 formed neurospheres on day 7. The neurosphere formation rates then significantly increased, and 50.2% ± 4.167 from GL261 and 53.0% ± 3.391 from U87 (d14 spheres/d3 surviving cells) formed neurospheres after hypoxia exposure for 14 d. More surprisingly, the sphere rates eventually reached 93.1% ± 5.541 from GL261 and 95.6% ± 2.665 from U87 (d21 spheres/d3 surviving cells) under hypoxic conditions for 21 days. However, in normoxia or hyperoxia, no distinct neurospheres formed, and most cells remained a single cell and eventually died (^*^ *P* < 0.05; ^**^ *P* < 0.05; ^#^ *P* > 0.05, One-way ANOVA). (**D,E**) Newly formed GL261 neurospheres highly expressed the stem cell markers CD133, CD15 and NESTIN and the chemoresistance-related proteins ABCG2 and MGMT (^*^ *P* < 0.05, Paired-samples T Test). (**F**) Trypan blue indicated there were no cells with blue-staining in the cytoplasm. (**G**) Asymmetric division was detected for newly formed neurospheres, and the results indicated cells in neurospheres maintained growth in suspension with a sphere morphology when cultivated in GSC medium for 5 d; however, adherent growth with a differentiation style was induced after 10%FBS administration.

We detected the influence of oxygen on the growth of CD133^−^CD15^−^NESTIN^−^ glioma cells. One CD133^−^CD15^−^NESTIN^−^ cell was plated in each well of 96-well plates, and these plates were cultured in different oxygen levels. The results showed a single GL261 and U87 CD133^−^CD15^−^NESTIN^−^ cell grew well after seeding, and approximately 75% of the cells (d3 viable cells/d0 seeding cells) survived after different oxygen exposures for 3 days without significant differences (Fig. 3A,B; Supplementary Table S1). We subsequently observed and recorded the cell growth at d3, 7, 14 and 21 under different oxygen levels, and the results indicated neurospheres began to form after 1%O_2_ exposure for 3 d. Approximately 20% of the surviving cells (d7 spheres/d3 surviving cells) from GL261 and U87 formed neurospheres after 7 d of exposure in 1%O_2_. The neurosphere rates then significantly increased, and 50.2% ± 4.167 from GL261 and 53.0% ± 3.391 from U87 (d14 spheres/d3 surviving cells) surviving cells formed neurospheres after hypoxia exposure for 14 d. More surprisingly, the sphere rates eventually reached 93.1% ± 5.541 from GL261 and 95.6% ± 2.665 from U87 (d21 spheres/d3 surviving cells) under hypoxic conditions for 21 d. However, under normoxia or hyperoxia, most cells remained a single cell and eventually died (Fig. 3A,C; Supplementary Table S1).

### Newly formed neurospheres in hypoxic environment presented the features of GSCs

Newly formed neurospheres from GL261 CD133^−^CD15^−^NESTIN^−^ cells in hypoxia highly expressed the stem cell markers CD133, CD15 and NESTIN according to the immunofluorescence results. Furthermore, the chemoresistance-related proteins ABCG2 and MGMT significantly increased in these neurospheres (Fig. 3D). RT-qPCR and western blot assays also showed that CD133, CD15, NESTIN, ABCG2 and MGMT in GL261 newly formed neurospheres exhibited higher expression levels compared with control CD133^−^CD15^−^NESTIN^−^ cells cultured in normoxia (Fig. 3E; Supplementary Figure S2). Similar results were obtained in U87 newly formed neurospheres (data not shown). Trypan blue staining was used to determine whether these cells in neurospheres were alive, and we found there were no cells with blue staining in the cytoplasm (Fig. 3F). Asymmetric division, one of the most important characteristics of GSCs^22^, was detected under 21%O_2_ environment in GSC medium (DMEM/F12 + EGF + FGF2 + B27) or differentiated medium (DMEM/F12 + 10%FBS) for newly formed neurospheres. The results showed the cells maintained growth in suspension with a sphere morphology when cultivated in GSC medium for 5 d; however, adherent growth with a differentiation style was induced after 10%FBS administration (Fig. 3G). Besides, *in vivo*, the cells digested by GL261-luc neurospheres showed higher tumorigenic when implanting into C57 mice brain than control GL261-luc CD133^−^CD15^−^NESTIN^−^ cells with the same treatments (Supplementary Figure S9).

### CD133^+^CD15^+^NESTIN^+^ neurospheres formed in 1%O_2_ presented different growth characteristics under 1%O_2_, 21%O_2_ and 95%O_2_

**Figure 4:**
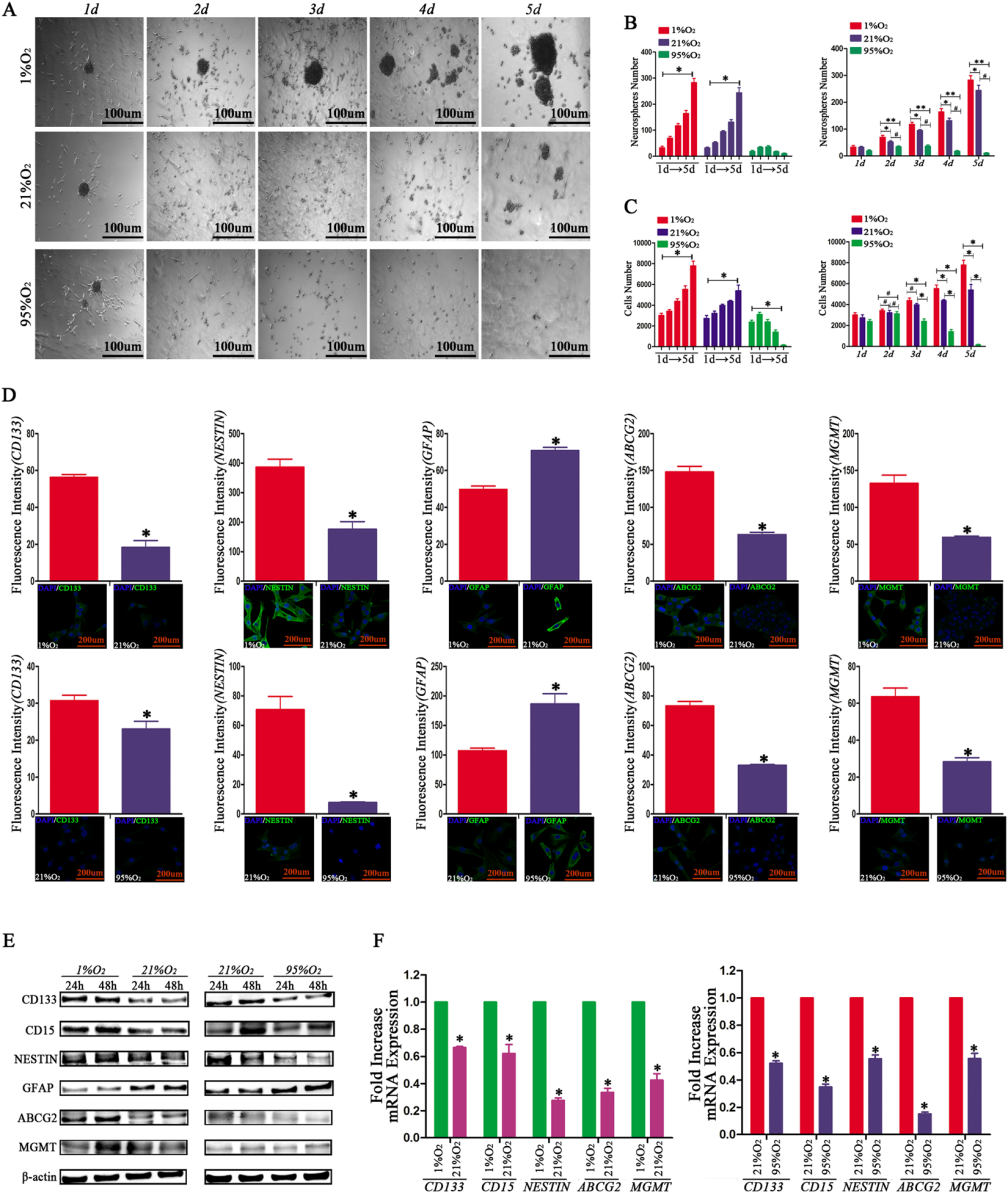
Neurospheres formed in 1%O_2_ presented different growth styles under different oxygen levels. (**A**) U87 CD133^+^CD15^+^NESTIN^+^ neurospheres maintained growth in suspension with a sphere morphology under 1%O_2_ conditions; under 21%O_2_, some neurospheres also existed after exposure for 5 d. However, there were no neurospheres after 95%O_2_ exposure for 5 d. (**B**) The number of neurospheres increased in a time-dependent manner from hypoxia exposure for 1 to 5 d; compared with 21%O_2_, the number of neurospheres in 1%O_2_ was substantially increased, whereas under hyperoxic conditions, few neurospheres were detected (^*^ *P* < 0.05; ^**^ *P* < 0.05; ^#^ *P* < 0.05, One-sample T Test, One-way ANOVA). (**C**) There were no differences among the different oxygen levels on day 2 for the cell number of U87 CD133^+^CD15^+^NESTIN^+^ neurospheres. After three days, the number of cells under 95%O_2_ gradually decreased. In contrast, the cell numbers in 1%O_2_ and 21%O_2_ increased in a time-dependent manner, and the cell proliferation rate of cells in 1%O_2_ was faster than that of cells in 21%O_2_ (^*^ *P* < 0.05; ^#^ *P* > 0.05, One-sample T Test, One-way ANOVA). (**D–F**) Hypoxia promoted the expression levels of CD133, CD15, NESTIN, ABCG2 and MGMT and inhibited GFAP for U87 CD133^+^CD15^+^NESTIN^+^ stem cells. In contrast, hyperoxia presented the opposite effects, which resulted in decreases of CD133, CD15, NESTIN, ABCG2 and MGMT and an increase of GFAP (^*^ *P* < 0.05, Paired-samples T Test).

We cultured U87 CD133^+^CD15^+^NESTIN^+^ neurospheres in DMEM/F12 + 1%FBS in 1%O_2_, 21%O_2_ and 95%O_2_. The U87 CD133^+^CD15^+^NESTIN^+^ neurospheres continued to grow in suspension with a sphere morphology in 1%O_2_; moreover, the number of neurospheres increased in a time-dependent manner in hypoxia exposure from 1 to 5 d. In 21%O_2_, some neurospheres also persisted after exposure for 5 d; however, the number of neurospheres was substantially fewer than those observed in 1%O_2_. Furthermore, the size of the neurospheres under 21%O_2_ was substantially smaller. However, in 95%O_2_, no neurospheres formed after 5d of exposure (Fig. 4A,B). With respect to the cell numbers of U87 CD133^+^CD15^+^NESTIN^+^ neurospheres after cultivation with different oxygen levels, we found there were no differences among 1%O_2_, 21%O_2_ and 95%O_2_ on d2. After three days, the cell numbers in 1%O_2_ and 21%O_2_ increased in a time-dependent manner. In contrast, the number of cells under 95%O_2_ gradually decreased (Fig. 4C).

### Protein expression in CD133^+^CD15^+^NESTIN^+^ neurospheres exposed to 1%O_2_, 21%O_2_ and 95%O_2_

U87 CD133^+^CD15^+^NESTIN^+^ cells digested from newly formed neurospheres highly expressed CD133, NESTIN, ABCG2 and MGMT after 1%O_2_ exposure for 24 h. Moreover, hypoxia inhibited GFAP expression. In contrast, there was reduced CD133, NESTIN, ABCG2 and MGMT expression in U87 CD133^+^CD15^+^NESTIN^+^ cells exposed to 21%O_2_ compared with those exposed to hypoxia. However, GFAP expression increased in cells exposed to 21%O_2_. We subsequently compared the expression of these proteins in U87 CD133^+^CD15^+^NESTIN^+^ cells between 21%O_2_ and 95%O_2_ and determined that there was increased GFAP expression in cells exposed to 95%O_2_. However, the expression of CD133, NESTIN, ABCG2 and MGMT significantly decreased after exposure to 95%O_2_ for 24 h (Fig. 4D). Western blot assays indicated similar results (Fig. 4E; Supplementary Figure S3A,B). Furthermore, western blotting showed that the expression of CD133, CD15, NESTIN, ABCG2 and MGMT increased in cells exposed to 1%O_2_ in a time-dependent manner from 24 to 48 h. However, in the 21%O_2_ and 95%O_2_ environments, these proteins remained stable or decreased in a time-dependent manner. In contrast, GFAP expression significantly increased in cells exposed to 95%O_2_ for 48 h compared with cells exposed to the same treatment for 24 h. However, in 1%O_2_ or 21%O_2_, the expression remained stable (Fig. 4E; Supplementary Figure S3C,D). RT-qPCR indicated the same results that hypoxia maintained or promoted the expression of CD133, CD15, NESTIN, ABCG2 and MGMT in U87 CD133^+^CD15^+^NESTIN^+^ cells (Fig. 4F).

### Cell cycle, apoptosis and IC50 detection of CD133^−^/CD15^−^/NESTIN^−^ glioma cells under different oxygen levels

**Figure 5:**
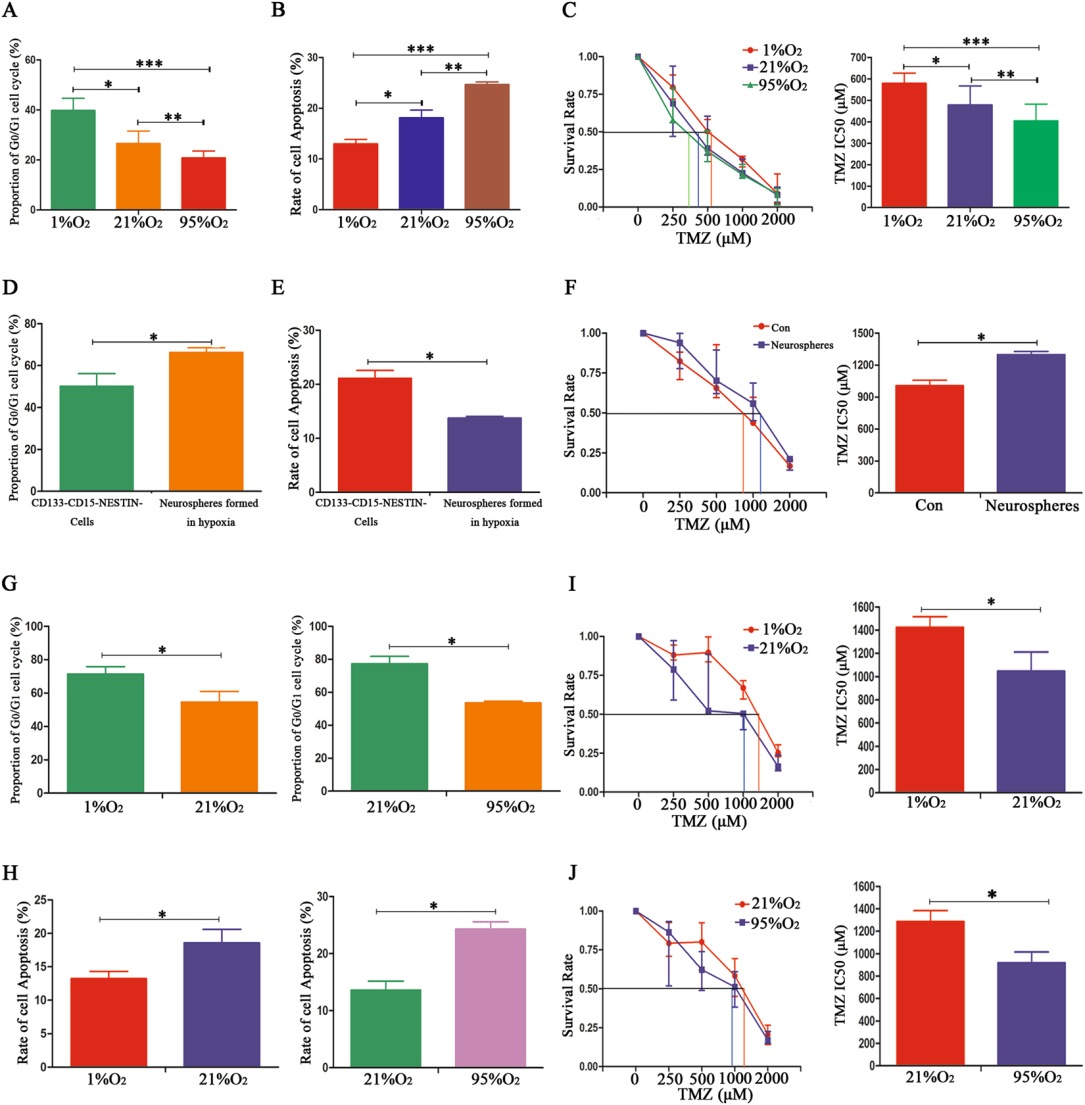
Cell cycle, apoptosis and IC50 detection for glioma cells under different oxygen levels. (**A**) U87 CD133^−^/CD15^−^/NESTIN^−^ cells arrested in G_0/_G_1_ under 1%O_2_ conditions compared with 21% O_2_ and 95% O_2_; a normoxic environment promoted more cells arrested in G_0_/G_1_ compared with 95%O_2_ (^*^ *P* < 0.05; ^**^ *P* < 0.05; ^***^ *P* < 0.05, One-way ANOVA). (**B**) The lowest apoptosis rate was identified in the 1%O_2_ microenvironment for U87 CD133^−^/CD15^−^/NESTIN^−^ cells after TMZ (100 μM) treatments, and the cells cultivated in 95%O_2_ had the highest cell apoptosis rate (^*^ *P* < 0.05; ^**^ *P* < 0.05; ^***^ *P* < 0.05, One-way ANOVA). (**C**) Compared with cells in 95%O_2_, there were higher mean IC50 values under 21%O_2_ for U87 CD133^−^/CD15^−^/NESTIN^−^ cells after TMZ treatments, and the mean IC50 values significantly increased in 1%O_2_ compared with 21%O_2_. The survival rates of U87 CD133^−^/CD15^−^/NESTIN^−^ cells after TMZ treatments demonstrated a decrease after an oxygen level increase (^*^ *P* < 0.05; ^**^ *P* < 0.05; ^***^ *P* < 0.05, One-way ANOVA). (**D**) U87 CD133^+^/CD15^+^/NESTIN^+^ neurospheres arrested the cell cycle in G_0_/G_1_ (^*^ *P* < 0.05, Paired-samples T Test). (**E**) A lower apoptosis rate was identified for U87 CD133^+^/CD15^+^/NESTIN^+^ neurospheres after TMZ (100 μM) treatments for 48 h than differentiated CD133^−^/CD15^−^/NESTIN^−^ cells with the same TMZ treatments (^*^ *P* < 0.05, Paired-samples T Test). (**F**) GL261 CD133^+^/CD15^+^/NESTIN^+^ neurospheres had a higher survival rate after TMZ exposure than CD133^−^/CD15^−^/NESTIN^−^ cells. The mean IC50 values of GL261 CD133^+^/CD15^+^/NESTIN^+^ neurospheres were 1.4-fold higher than differentiated CD133^−^/CD15^−^/NESTIN^−^ cells (^*^ *P* < 0.05, Paired-samples T Test). (**G**) U87 CD133^+^/CD15^+^/NESTIN^+^ cells arrested in the G_0_/G_1_ state in 1%O_2_ conditions compared with normoxia; there were more arrested in G_0_/G_1_ in normoxic compared with hyperoxic environments (^*^ *P* < 0.05, Paired-samples T Test). (**H**) U87 CD133^+^/CD15^+^/NESTIN^+^ cells treated with TMZ (100 μM) exhibited a lower apoptosis rate in the hypoxic environment than normoxia or hyperoxia (^*^ *P* < 0.05, Paired-samples T Test). (**I**) U87 CD133^+^/CD15^+^/NESTIN^+^ cells in 1%O_2_ conditions had a higher survival rate after TMZ treatment, and the mean IC50 value was approximately 1.3-fold higher than the control normoxic treatment cells (^*^ *P* < 0.05, Paired-samples T Test). (**J**) Hyperoxia had opposite effects for U87 CD133^+^/CD15^+^/NESTIN^+^ cells treated with TMZ, which exhibited a lower survival rate and IC50 values (^*^ *P* < 0.05, Paired-samples T Test).

Differentiated U87 and GL261 CD133^−^/CD15^−^/NESTIN^−^ cells were cultured in various oxygen levels for 48 h to detect the cell cycle. The results indicated that U87 CD133^−^/CD15^−^/NESTIN^−^ cells arrested the cell cycle in G_0_/G_1_ under 1%O_2_ conditions compared with those grown in 21%O_2_ and 95%O_2_; moreover, compared with 95%O_2_, there were more cells arrested in G_0_/G_1_ if the cells were cultured in 21%O_2_ (Fig. 5A). GL261 CD133^−^/CD15^−^/NESTIN^−^ cells exhibited the same results (data not shown). We subsequently added TMZ (100 μM) to these U87 and GL261 CD133^−^/CD15^−^/NESTIN^−^ cells and cultured them under different oxygen levels for an additional 48 h. The results showed that both GL261 and U87 CD133^−^/CD15^−^/NESTIN^−^ cells presented the lowest apoptosis rate when growth in 1%O_2_, and the cells cultivated in 95%O_2_ had the highest apoptosis rate (Fig. 5B). Furthermore, we analyzed the IC50 values and their respective ratios among cells grown in 1%O_2_, 21%O_2_ and 95%O_2_. Compared with the cells under 95%O_2_, the IC50 for the GL261 CD133^−^/CD15^−^/NESTIN^−^ cells increased 1.2-fold in 21%O_2_ and 1.4-fold in 1%O_2_. Similarly, the IC50 for the U87 CD133^−^/CD15^−^/NESTIN^−^ cells also increased 1.1-fold in 21%O_2_ and 1.2-fold in 1%O_2_ compared with cells under 95%O_2_. The difference between 21%O_2_ and 1%O_2_ was significant, which indicated there was a higher IC50 in the 1%O_2_ conditions. Overall, the IC50 and survival rates were highest for GL261 and U87 CD133^−^/CD15^−^/NESTIN^−^ cells under hypoxia, and the lowest IC50 and survival rates were identified in cells grown in 95%O_2_ (Fig. 5C; Supplementary Table S2A).

### Cell cycle, apoptosis and IC50 detection of CD133^+^/CD15^+^/NESTIN^+^ glioma neurospheres formed in 1%O_2_

As one of the most important features of GSCs, we determined whether the GL261 and U87 neurospheres that formed in 1%O_2_ were cycle arrested and chemoresistant to TMZ. First, flow cytometry indicated there were more cells arrested in G_0_/G_1_ with rates greater than 60% for U87 CD133^+^/CD15^+^/NESTIN^+^ neurospheres, which demonstrated a significant difference compared with CD133^−^/CD15^−^/NESTIN^−^ cells (Fig. 5D). A cell apoptosis examination indicated that U87 CD133^+^/CD15^+^/NESTIN^+^ neurospheres exhibited a lower apoptosis rate after exposure to TMZ (100 μM) for 48 h than CD133^−^/CD15^−^/NESTIN^−^ cells with the same TMZ treatment (Fig. 5E). Moreover, compared with GL261 CD133^−^/CD15^−^/NESTIN^−^ cells, CD133^+^/CD15^+^/NESTIN^+^ neurospheres after exposure to TMZ had a higher survival rate, in which the IC50 reached 1298.898 μM, which was approximately 1.4-fold higher than the value of 973.703 μM for control cells (Fig. 5F; Supplementary Table S2B). Similar results were obtained for U87 CD133^+^/CD15^+^/NESTIN^+^ neurospheres formed in 1%O_2_ (Supplementary Table S2B).

### Cell cycle, apoptosis and IC50 detection of CD133^+^/CD15^+^/NESTIN^+^ glioma neurospheres under different oxygen levels

CD133^+^/CD15^+^/NESTIN^+^ glioma cells formed from CD133^−^/CD15^−^/NESTIN^−^ cells after hypoxia treatment for 14 d were cultured in 1%O_2_, 21%O_2_ and 95%O_2_ for 48 h, and the changes in the cell cycle were subsequently detected. Compared with the cells in 21%O_2_, more U87 CD133^+^/CD15^+^/NESTIN^+^ cells arrested in the G_0_/G_1_ state in 1%O_2_ (Fig. 5G). TMZ (100 μM) was subsequently added to these cells, which were cultured for an additional 48 h, followed by cell apoptosis detection. We determined there was a lower cell apoptosis rate in hypoxia than normoxia (Fig. 5H). Furthermore, the results indicated that U87 CD133^+^/CD15^+^/NESTIN^+^ cells in 1%O_2_ had a higher survival rate after TMZ treatment, and the IC50 value was approximately 1.3-fold higher than that of control normoxia-treated cells (Fig. 5I; Supplementary Table S2C). Hyperoxia presented an opposite effect, which resulted in the lowest rate of G_0_/G_1_ and the highest apoptosis rate (Fig. 5G,H, J; Supplementary Table S2C).

### Higher levels of HIF1α in hypoxia, and hyperoxia inhibited HIF1α

**Figure 6:**
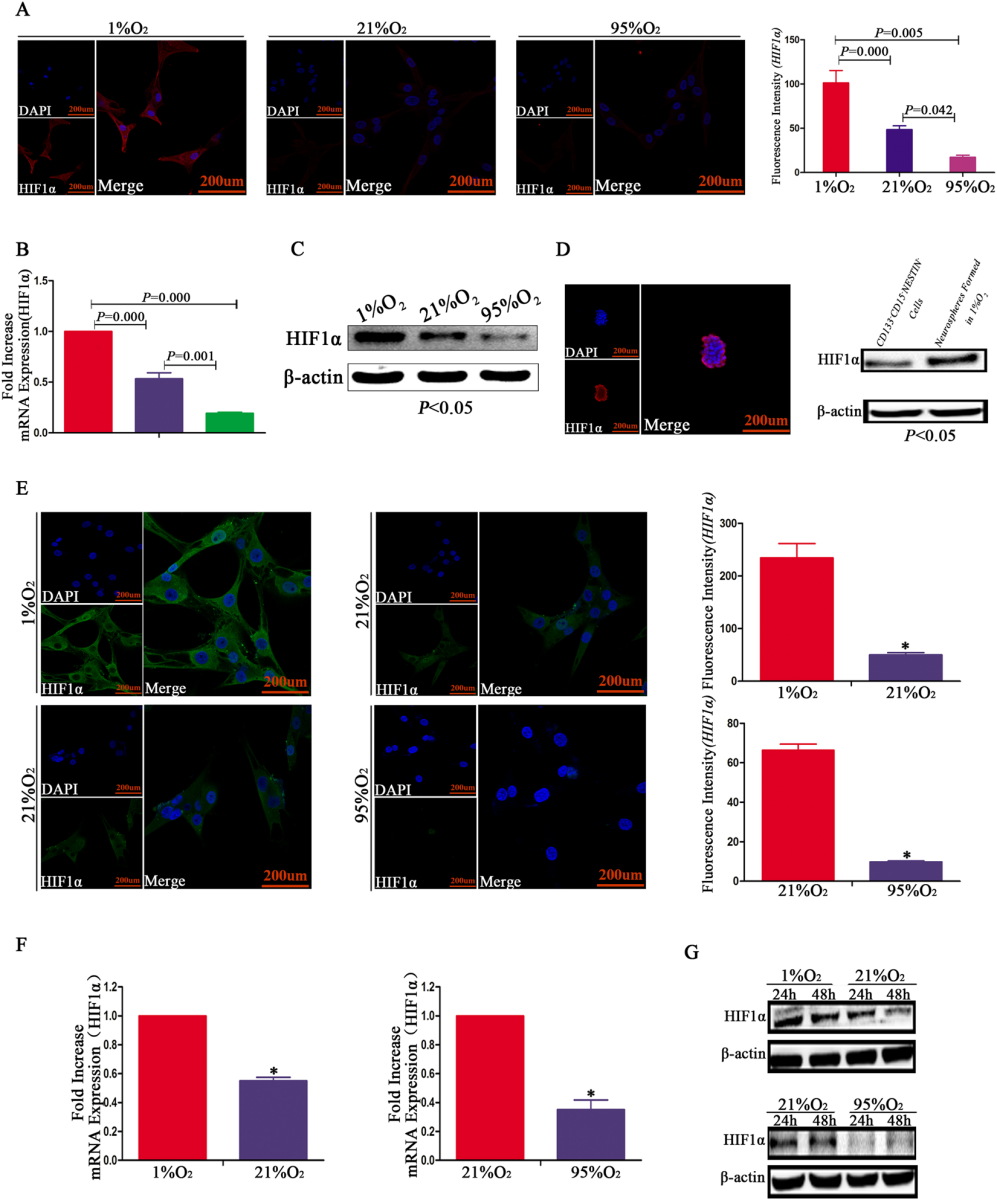
Higher levels of HIF1α under hypoxic conditions, and hyperoxia inhibited HIF1α expression. (**A**) An increased expression of HIF1α for U87 CD133^−^/CD15^−^/NESTIN^−^ cells in 1%O_2_ conditions, which was predominately located in the cell cytoplasm; however, the HIF1α levels significantly decreased after exposure in 21%O_2_ or 95%O_2_ (*P* < 0.05, One-way ANOVA). (**B**) RT-qPCR indicated a 2-fold increase of HIF1α mRNA levels for U87 CD133^−^/CD15^−^/NESTIN^−^ cells in the 1%O_2_ environment compared with normoxia; the expression of HIF1α was the lowest after hyperoxia exposure (*P* < 0.05, One-way ANOVA). (**C**) HIF1α expression was the highest in 1%O_2_ conditions, and the lowest levels of HIF1α were identified for U87 CD133^−^/CD15^−^/NESTIN^−^ cells in 95%O_2_. (**D**) GL261 neurospheres formed in 1%O_2_ conditions highly expressed HIF1α. (**E–G**) An enhancement of HIF1α for newly formed U87 neurospheres in hypoxia was identified compared with normoxia and hyperoxia (^*^ *P* < 0.05, Paired-samples T Test).

U87 CD133^−^/CD15^−^/NESTIN^−^ cells were cultivated in different oxygen levels for 24 h, and the immunofluorescence results indicated the highest HIF1α expression occurred in cells exposed to 1%O_2_, and no HIF1α was detected in cells exposed to 95%O_2_. However, the dramatic difference from traditional studies was that most HIF1α was located in the cell cytoplasm, with a limited amount located in the cell nucleus. Under normoxic conditions, some HIF1α was detected in U87 CD133^−^/CD15^−^/NESTIN^−^ cells; HIF1a expression was decreased 2-fold under these conditions compared with the cells exposed to hypoxia and increased 2-fold compared with those exposed to a hyperoxic environment (Fig. 6A). Similar results were obtained for RT-qPCR, and HIF1α expression was significantly decreased in U87 CD133^−^/CD15^−^/NESTIN^−^ cells with an increase in oxygen-level dependence (Fig. 6B). Western blotting indicated the highest levels of HIF1α were observed in cells exposed to 1%O_2_, and HIF1α expression decreased when the oxygen level increased for U87 CD133^−^/CD15^−^/NESTIN^−^ cells (Fig. 6C). Furthermore, GL261 neurospheres formed in 1%O_2_ also highly expressed HIF1α (Fig. 6D). We subsequently digested U87 neurospheres and prepared cell suspensions and cultured them in different oxygen levels for 24 h. According to immunofluorescence, HIF1α expression in U87 neurospheres cultured in 1%O_2_ was approximately 5.5-fold higher than that of cells exposed to normoxia. Furthermore, HIF1α significantly decreased after hyperoxia exposure compared with the cells cultured in 21%O_2_ (Fig. 6E). Similar results were obtained via RT-qPCR and western blot (Fig. 6F,G, Supplementary Figure S4A-B).

### Influences of HIF1α on protein expression and cell apoptosis

**Figure 7:**
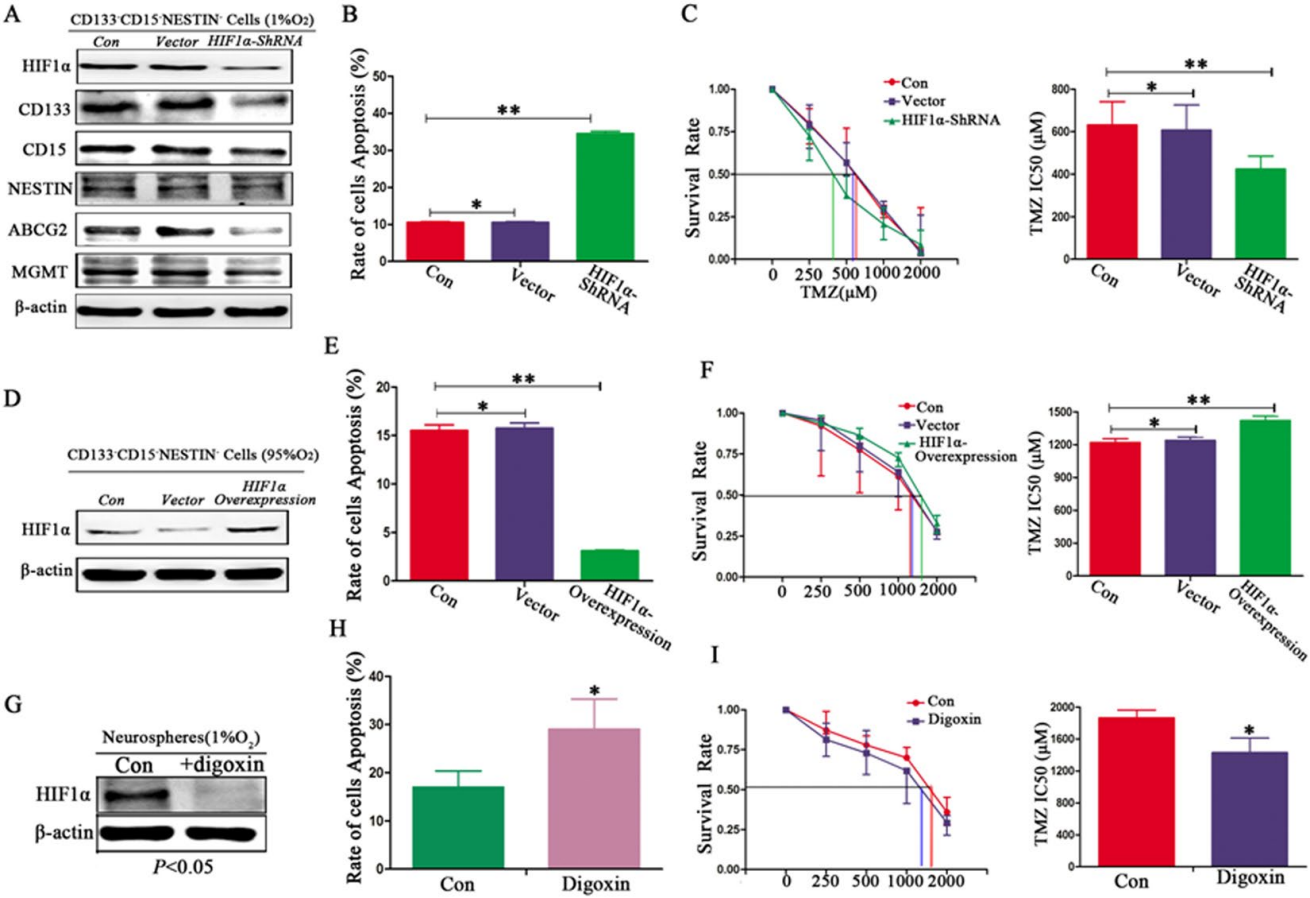
Influences of HIF1α on protein expression and cell apoptosis. (**A**) After interfering with HIF1α expression using ShRNA, HIF1α levels were substantially lower, and the levels of CD133, CD15, NESTIN, ABCG2 and MGMT for HIF1α-silenced U87 CD133^−^/CD15^−^/NESTIN^−^ cells were significantly decreased compared with the control and vector-U87 CD133^−^/CD15^−^/NESTIN^−^ cells under 1%O_2_ conditions. (**B**) HIF1α-silenced U87 CD133^−^/CD15^−^/NESTIN^−^ cells presented an approximately 3.6-fold higher apoptosis rate than the control and U87 vector cells in the hypoxic environment after TMZ (100 μM) treatments (^*^ *P* > 0.05; ^**^ *P* < 0.05, One-way ANOVA). (**C**) HIF1α-silenced U87 CD133^−^/CD15^−^/NESTIN^−^ cells exhibited a decreased survival rate with a lower IC50 in the hypoxic environment (^*^ *P* > 0.05; ^**^ *P* < 0.05, One-way ANOVA). (**D**) HIF1α was over-expressed for U87 CD133^−^/CD15^−^/NESTIN^−^ cells in a 95%O_2_ environment, and western blotting indicated that HIF1α expression was significantly increased. (**E**) Decreased apoptosis was noted for HIF1α-over-expressed U87 CD133^−^/CD15^−^/NESTIN^−^ cells compared with control and vector cells in the hyperoxic environment after TMZ (100 μM) treatments (^*^ *P* > 0.05; ^**^ *P* < 0.05, One-way ANOVA). (**F**) HIF1α-over-expressed U87 CD133^−^/CD15^−^/NESTIN^−^ cells exhibited an increased survival rate with a higher IC50 in the hyperoxic environment (^*^ *P* > 0.05; ^**^ *P* < 0.05, One-way ANOVA). (**G**) Western blot indicated there was reduced HIF1α expression for U87 CD133^+^/CD15^+^/NESTIN^+^ neurospheres after digoxin (100 μM) treatment. (**H**) Cell apoptosis occurred at a higher rate in U87 CD133^+^/CD15^+^/NESTIN^+^ neurospheres treated with digoxin after TMZ (100 μM) treatment in the hypoxic environment than in cells without digoxin (^*^ *P* < 0.05, Paired-samples T Test). (**I**) Lower survival rates and 2-fold decrease of IC50 for U87 CD133^+^/CD15^+^/NESTIN^+^ neurospheres treated with digoxin after TMZ treatments were detected in the hypoxic environment (^*^ *P* < 0.05, Paired-samples T Test).

We subsequently knocked down the HIF1α gene through Sh-RNA for U87 CD133^−^/CD15^−^/NESTIN^−^ cells, and western blotting assays indicated that the HIF1α expression was lost compared with the control and vector cells in 1%O_2_ (Fig. 7A). We cultured these cells for 48 h in hypoxia and determined that the expression levels of CD133, CD15, NESTIN, ABCG2 and MGMT were significantly decreased for the ShRNA U87 CD133^−^/CD15^−^/NESTIN^−^ cells compared with the control and vector cells (Fig. 7A; Supplementary Figure S5A). Moreover, in hypoxic conditions, U87 CD133^−^/CD15^−^/NESTIN^−^ cells with HIF1α interference presented a decrease in neurosphere formation at 21 d, with its rate at approximately 66.50 ± 3.10% However, the neurosphere formation rates were greater than 90% for both the control and U87 vector cells, and the size of the neurospheres was substantially larger than that for U87 HIF1α silencing cells under hypoxic conditions (Supplementary Figure S6A,B, Table S3). Furthermore, the ShRNA U87 CD133^−^/CD15^−^/NESTIN^−^ cells also presented a higher apoptosis rate, which was increased approximately 3.6-fold compared with the control and U87 vector cells (Fig. 7B). The survival rates decreased for the ShRNA U87 CD133^−^/CD15^−^/NESTIN^−^ cells, and the IC50 values decreased (Fig. 7C; Supplementary Table S2D). Furthermore, we over-expressed HIF1α in U87 CD133^−^/CD15^−^/NESTIN^−^ cells in 95%O_2_ (Fig. 7D; Supplementary Figure S5B), which resulted in lower apoptosis rates and an increased IC50 (Fig. 7E,F; Supplementary Table S2E). Digoxin was used to inhibit HIF1α for U87 CD133^+^/CD15^+^/NESTIN^+^ neurospheres formed in 1%O_2_ (Fig. 7G). We cultured these GSCs in a hypoxic environment with digoxin and added TMZ (100 μM) into the cell suspension for 48 h, which resulted in higher cell apoptosis (Fig. 7H), a lower survival rate and a 2-fold decrease of IC50 than the control cells without digoxin treatments (Fig. 7I; Supplementary Table S2F).

## Discussion

The main focus of this study was to investigate the mechanism of glioma cell chemosensitivity in different oxygen levels. Traditional studies have ascribed the chemoresistance of glioma to GSCs as a result of the higher expression of chemoresistance-related proteins, such as ABCG2 and MGMT^3, 8, 11, 23–25^. However, other types of glioma cells exist, which we referred to as normal differentiated glioma cells. In contrast to GSCs, differentiated glioma cells are more sensitive to chemotherapy drugs, such as TMZ^6, 7, 11^. Conventional chemotherapy only kills differentiated glioma cells and leaves GSCs intact, which results in chemoresistant glioma^26^. Furthermore, it has been reported that the chemoresistance of GSCs is markedly decreased if GSCs differentiate into normal differentiation glioma cells that exhibit specific differentiation hallmarks, such as GFAP^7, 11, 27^. Therefore, we conclude that the relationship between GSCs and normal differentiated glioma cells is of vital importance to glioma cell chemosensitivity.

Hypoxia in glioma also plays an important role in the maintenance of GSC stemness, which thus promotes glioma chemoresistance^20, 28^. Recently, researchers have cultivated unsorted glioma cells with stem cell medium in a hypoxic environment, and their results indicated a higher expression of stem cell markers, such as CD133, sox-2 and oct-4^13, 14, 29^. There is no doubt that the enhanced expression of CD133 may be due to the proliferation of CD133^+^ cells that exist in the unsorted cells; however, another possibility exists that these enhanced CD133 cells originate from differentiated cells through dedifferentiation. Actually, this phenomenon has been proved by our team recently, in this study we found cancer stem cells, including glioma, hepatom and lung cancer, can be induced through dedifferentiation in hypoxia^30, 31^. In contrast, as an effective way to alleviate a hypoxic environment, hyperoxia promotes the sensitivity of glioma cells to TMZ by decreasing the expression of MGMT, ABCG2 and Ki67 and inducing a higher cell apoptosis rate, resulting in an extended survival time for glioma patients^15, 16, 18, 19, 32^. However, the detailed mechanism requires further investigation. For normal stem cells, such as neural stem cells (NSCs) and mesenchymal stem cells (MSCs), researchers have indicated that hyperoxia promotes differentiation and inhibits the expression of stem cell markers^32–34^. Unfortunately, to date, the influence of hyperoxia on glioma stem cells has not been clearly understood. Therefore, these studies suggest GSCs may be induced through dedifferentiation under hypoxic conditions, and hypoxia also contributes to the maintenance of stemness, which thus results in chemoresistance. In contrast, differentiated glioma cells may have differentiated from GSCs under hyperoxic conditions, and hyperoxia inhibits the dedifferentiation process.

Because of the query of CD133 as a GSC marker^2, 10, 35^, we combined CD133 with CD15^36^ and NESTIN^2, 37, 38^ as GSC markers to improve the validity of our study, and GFAP^7, 11, 27^ was used as the differentiated marker of glioma cells according to traditional studies. Differentiated CD133^−^/CD15^−^/NESTIN^−^ glioma cells were initially sorted through magnetic cell sorting (MACS) and cultured under 1%O_2_, 21%O_2_ and 95%O_2_ conditions. After 1%O_2_ exposure, hypoxia promoted the expression of GSC markers and inhibited GFAP. However, hyperoxia presented the opposite effects and resulted in an inhibition of these stem cell markers with an increase in GFAP. Here, we should note that the enhanced CD133, CD15 and NESTIN may originate from CD133^+^/CD15^+^/NESTIN^+^ GSCs as a result of contamination when sorting CD133^−^/CD15^−^/NESTIN^−^ glioma cells^35^. To avoid this possibility, we detected the rate of newly formed neurospheres by a single CD133^−^/CD15^−^/NESTIN^−^ glioma cell; the results indicated that more than 95% glioma cells (d21 neurospheres/d3 viable cells) formed neurospheres with an increased expression of CD133, CD15 and NESTIN after hypoxia exposure for 21 d; however, the cells cultured in normoxia or hyperoxia maintained a single cell and eventually died. Some neurospheres that formed in 1%O_2_ originated from CD133^+^/CD15^+^/NESTIN^+^ GSCs; however, the proportion of GSCs in glioma was less than 20%^2, 39^. Thus, the other 75% (75% = 95–20%) of newly formed neurospheres should be from CD133^−^/CD15^−^/NESTIN^−^ glioma cells through dedifferentiation. Furthermore, the CD133^−^/CD15^−^/NESTIN^−^ cells were sorted through MACS at least three times; as a result, the proportion of CD133^+^/CD15^+^/NESTIN^+^ GSCs should be less than 20%.

Traditional studies have indicated that the chemoresistance-related proteins ABCG2 and MGMT are highly expressed in GSCs^3, 23, 25^. This is one of the reasons why GSCs have a higher chemoresistance than non-GSCs. To investigate whether this theory is valid, we detected cell apoptosis for sorted CD133^+^/CD15^+^/NESTIN^+^ GSCs after TMZ treatments, and the results indicated that GSCs exhibited a lower apoptosis rate. In our study, we determined that the levels of ABCG2 and MGMT were significantly increased, in addition to the high expression of stem cell markers for differentiated CD133^−^/CD15^−^/NESTIN^−^ cells after hypoxia treatment; moreover, the expression levels of ABCG2 and MGMT decreased in hyperoxia. Newly formed neurospheres in hypoxia also highly expressed ABCG2 and MGMT. We subsequently added TMZ to the cell suspensions and cultured them in different oxygen levels for an additional 48 h; the differentiated CD133^−^/CD15^−^/NESTIN^−^ cells under hypoxic conditions had the lowest apoptosis rate compared with those in normoxia and hyperoxia. The mean IC50 values presented 1.2-fold higher in hypoxia than hyperoxia. According to these results, we conclude that hypoxia induces the formation of GSCs through dedifferentiation from differentiated cells, which results in resistance to chemotherapy drugs, such as TMZ. In contrast, hyperoxia inhibits the dedifferentiation processes, which leads to the sensitization to TMZ.

Researchers have indicated that hypoxia contributes to GSC stemness^11, 12, 28^. For example, in 2009, Li *et al*.^40^ cultivated GSCs in hypoxic conditions and identified a significant enhancement of stem cell activity with a higher expression of CD133 and an easier formation of neurospheres. Therefore, we investigated the influence of different oxygen levels on CD133^+^/CD15^+^/NESTIN^+^ neurospheres. The results showed in hypoxic conditions, these cells maintained growth in sphere morphology with an increased expression of CD133, CD15 and NESTIN and a decrease of GFAP. In contrast, hyperoxia significantly enhanced the GFAP expression and inhibited CD133, CD15 and NESTIN. Furthermore, under hyperoxic conditions, the cells maintained adherent growth, and there were few neurospheres. We also demonstrated the levels of ABCG2 and MGMT increased under hypoxic conditions, and a significant decrease was identified after hyperoxia treatments. The apoptosis detection indicated these CD133^+^/CD15^+^/NESTIN^+^ cells exhibited a higher apoptosis rate in hyperoxia, and the mean IC50 values were substantially lower. Thus, hypoxia maintains GSC stemness to sustain the resistance of glioma to TMZ; in contrast, hyperoxia promotes GSC differentiation, which effectively overrides glioma resistance to TMZ.

Studies have shown that HIF1α contributes to the maintenance of GSC stemness^11, 29^. Furthermore, HIF1α promotes a significant phenotypic shift towards an undifferentiated population through dedifferentiation from normal glioma cells after TMZ treatments^41^. HIF1α regulates the expression of ABCG2 and MGMT, which thus influences sensitivity to TMZ^11, 42^. HIF1α is influenced by oxygen levels; researchers have demonstrated that hypoxia enhances the stability of HIF1α, and HIF1α will be degraded if oxygen levels increase^43, 44^. Thus, we investigated whether HIF1α exerts an influence in our experiments. We initially identified HIF1α as highly expressed in 1%O_2_ in differentiated cells and GSCs. However, the HIF1α levels substantially decreased when the oxygen levels increased. Correspondingly, HIF1α silencing significantly inhibited the expression levels of CD133, CD15, NESTIN, ABCG2 and MGMT under hypoxic conditions. Moreover, HIF1α suppression induced cells to exit G_0_/G_1_ and induced greater levels of cell apoptosis. In contrast, an increased resistance of TMZ to glioma cells was demonstrated after HIF1α over-expression in 95%O_2_. Moreover, the mean IC50 values were enhanced after HIF1α over-expression in glioma cells compared with the control.

In summary, following an analysis of our results, we conclude that HIF1α facilitates the dedifferentiation of normal glioma cells and maintains GSC stemness to promote chemoresistance. These findings are consistent with those of Auffinger *et al*.^10^, who showed that GSCs may be induced through dedifferentiation following glioma cell treatment with TMZ. In contrast, hyperoxia exerts the opposite effects, in which chemosensitization is induced through the differentiation of GSCs, and an inhibition of dedifferentiation occurs as a result of the degradation of HIF1α. This study indicates that normal glioma cells and GSCs may be transformed through dedifferentiation and differentiation regulated by HIF1α under different oxygen levels, which therefore influence the sensitivity of glioma cells to chemotherapy. These findings provide a novel target for the treatment of glioma.

## Materials and Methods

### Cell isolation and Cell culture

GL261 and U87 cells were considered glioma cell lines. We used magnetic cell sorting (MACS; Miltenyi Biotech, Bergisch-Gladbach, Germany) to sort differentiated CD133^−^CD15^−^NESTIN^−^ glioma cells and CD133^+^CD15^+^NESTIN^+^ glioma stem cells^45^ and the detailed methods of MACS were shown in supplementary materials. GL261 and U87 cells were initially incubated in DMEM/F12 + 10%FBS medium at 21%O_2_ to maintain cell growth. The glioma cells were subsequently digested by 0.25% trypsin, centrifuged and suspended with DMEM/F12 + 10%FBS medium. Second, the cells were centrifuged again and re-suspended in PBS with 0.08%EDTA and 0.5%BSA (PBSE; 10^8^ cells/500 μl). Polyclonal rabbit anti-mouse or human CD133^+^ IgGs (Miltenyi Biotech, Germany) was added and maintained at 4 °C for 15 min. The cells were subsequently washed with PBS that contained 1%BSA and centrifuged again. The cells were suspended in PBSE (10^8^ cells/300 μl), and goat anti-rabbit IgG MicroBeads (Miltenyi Biotech, Germany) was added to the cell suspension and cultured at 10 °C. After 15 min, the cells were washed at least twice with PBSE. The cell number was counted, and the cells were suspended in 500 μl of PBSE. The cell suspension was poured into a column reservoir, which was placed by a miniMACS magnet and flushed with 500 μl of PBSE; unlabeled nonmagnetic CD133^−^ cells were collected in a culture flask. The cells retained on the magnet were CD133^+^ cells. We repeated these steps at least three times to purify the sorted cells. We subsequently used the same methods to sort CD15^−^ and NESTIN^−^ cells from sorted CD133^−^ cells. CD133^+^CD15^+^NESTIN^+^ glioma stem cells were also sorted as described. Sorted CD133^−^CD15^−^NESTIN^−^ glioma cells were cultured in DMEM/F12 + 10%FBS culture medium, and CD133^+^CD15^+^NESTIN^+^ glioma stem cells were cultured in stem cell medium (DMEM/F12 + EGF + FGF2 + B27).

### Oxygen treatment

Glioma cells were exposed to different oxygen levels (hypoxia, 1%O_2_; normoxia, 21%O_2_; hyperoxia, 95%O_2_) under normobaric conditions with or without TMZ. For hypoxic and normoxic treatments, cells were exposed to corresponding oxygen levels at all times^13^; for hyperoxic treatment, cells were exposed to 95%O_2_ for 60 min every 12 h^15, 16, 46^. CD133^−^CD15^−^NESTIN^−^ glioma cells were maintained in DMEM/F12 + 1%FBS. For CD133^+^CD15^+^NESTIN^+^ GSCs, cells were cultured in DMEM/F12 + 1%FBS when comparing the related changes between hypoxia and normoxia; when analyzing the difference between normoxia and hyperoxia, we cultivated the cells in DMEM/F12 + 10%FBS culture medium.

### Immunofluorescence staining

New neurospheres formed in hypoxia and glioma cells cultured under different oxygen levels for 24 h were examined for the expression of stem cell markers (CD133, CD15 and NESTIN), differentiated marker (GFAP) and chemoresistance proteins (ABCG2 and MGMT). After treatment, cells were collected and fixed with 4% paraformaldehyde for at least one day. The cells were subsequently washed with PBS and blocked using 10% serum in PBS with 0.5% Triton X-100 for 20 min. The primary antibodies against CD133, CD15, NESTIN, GFAP, ABCG2, MGMT and HIF1α (related information regarding the primary antibodies is presented in Supplementary Table S4) were added to the cell suspension at 4 °C for 24 h. The cells were subsequently washed with PBS for 5 min at least three times and incubated with appropriate fluorophore-labeled secondary antibodies for 1 h at 37 °C. A laser-scanning confocal microscope (LSM780, ZEISS, Germany) was used to obtain images.

### Western blot detection

The related protein expression was detected for neurospheres and glioma cells treated with different oxygen levels for 24 and 48 h. Cells were collected and subjected to SDS-PAGE, followed by transfer to nitrocellulose membranes. The cells were blocked with 5% milk and incubated with primary antibodies against β-actin, CD133, CD15, NESTIN, GFAP, ABCG2, MGMT and HIF1α (related information regarding the primary antibodies is presented in Supplementary Table S4) for 1 h. HRP-conjugated secondary antibodies were diluted at 1:10,000. Enhanced chemiluminescence was conducted for visualization. The semi-quantitative densitometry was calculated by Quanative-One software.

### Real-time quantitative polymerase chain reaction

Neurospheres and glioma cells treated with different oxygen levels for 12 h were assessed for mRNA levels, including CD133, CD15, NESTIN, GFAP, ABCG2, MGMT and HIF1α. The melting, denaturing and annealing temperatures were 94 °C, 5 min, 94 °C, 30 s and 57 °C, 30 s, respectively. The related primer sequences are shown in Supplementary Table S5.

### Flow cytometric analysis

Cells were cultured at different oxygen levels for 48 h, and the cell cycle was detected by flow cytometry (FCM); TMZ (100 μM) was subsequently added to the cell suspension, and the cells were cultured at different oxygen levels for an additional 48 h, followed by apoptosis detection. Neurospheres formed under hypoxia were also examined with regard to the cell cycle. Detailed steps of the detection of cell cycle and apoptosis were performed as follows. Cell cycle: cells were digested with 0.25% pancreatin and prepared as single cell suspension in 4 °C PBS. Then cells were suspended at 5 × 10^5^ cells/ml and fixed with ethanol 75% at 4 °C for 24 h. Cells were centrifugated and washed by PBS, and resuspended in 1 ml PI staining solution and incubated 30 min at 37 °C. Cell apoptosis: washed cells in 4 °C PBS and prepared single cell suspension. Counted cells at 1 × 10^6^ cells/ml and sucked up 100 μl suspension. Centrifugated and added 195 μl 0.05% trinatriumcitrate-dihydrate and 0.05% Triton X-100 and 5 μl Annexin V-FITC and incubated cells suspension 15 min. Centrifugated again and added 190 μl 0.05% trinatriumcitrate-dihydrate and 0.05% Triton X-100 and 10 μl PI and incubated cells suspension 20 min. Both samples of cell cycle and apoptosis were analysed by FACS (BD Accuri C6, Germany).

### IC50 detection

Cells were seeded into 96-well plates (5,000 cells/well) and cultivated under different oxygen levels for 48 h, followed by exposure in the absence or presence of TMZ (250, 500, 1,000 and 2,000 μM) for an additional 48 h. A total of 10 µl of CCK-8 was mixed with 90 µl of DMEM/F12 + 1%FBS medium, and the 100-µl suspensions were added to each well. After culturing the cell suspensions under different oxygen degrees for an additional 2 h, the OD value was measured with an ELISA reader at an absorbance of 450 nm (Varioskan Flash, Thermo Scientific, USA).

### Clonogenicity, trypan blue and asymmetric division assay

CD133^−^CD15^−^NESTIN^−^ glioma cells were digested, counted and diluted to 1,500 cells/1 ml DMEM/F12 + 10%FBS. A total of 1 µl of the medium was transferred to one well of a 96-well plate, which was pre-coated with 200 μl of serum-free DMEM/F12. Six 96-well plates were used and randomly divided into three groups; one group was cultured at 37 °C with 1%O_2_ and 5%CO_2_, the second group was cultivated under 21%O_2_ and the last group was incubated with 95%O_2_. The number of surviving cells was counted after exposure for 3 d, and the cell states were recorded at 0, 3, 7, 14 and 21 days (d). The cells were subsequently digested, and the cell suspension was prepared. One milliliter of trypan blue was added to the cell suspension. After three minutes, the cell color was determined under a microscope. We detected asymmetric division and washed and centrifuged the other cell suspension with PBS, following which the cells were transferred to two 24-well plates. One plate was cultured with stem cell medium (DMEM/F12 + EGF + FGF2 + B27), and the second plate was cultured with DMEM/F12 + 10%FBS. All plates were incubated at 37 °C with 21%O_2_ and 5%CO_2_, and the cell morphology was recorded at 1, 3 and 5 d. For CD133^+^CD15^+^NESTIN^+^ neurospheres formed under hypoxic conditions, we centrifuged and collected them. These neurospheres were randomly divided into three 24-well plates and cultivated under 1%O_2_, 21%O_2_ and 95%O_2_. The cell morphologies were recorded at days 1, 2, 3, 4 and 5.

### HIF1α interference assays

The HIF1α-ShRNA lentivirus and non-targeting control were obtained from Cyagen, Guangzhou. Approximately 10^5^ CD133^−^CD15^−^NESTIN^−^ cells were seeded into 6-well plates, and HIF1α-ShRNA-lentivirus (MOI = 1) was added into 6-well plates. After allowing for cell attachment for 24 h, 1 μg/ml of puromycin was added to 6-well plates, and the cell suspension was cultured for 48 h. These steps were repeated, and cells were collected after one week. Western blot was used to detect the HIF1α expression. Digoxin (TOCRIS, 4583) was used to decrease HIF1α for CD133^+^CD15^+^NESTIN^+^ glioma stem cells formed under hypoxic conditions. We initially dissolved the effective digoxin concentration to 100 nm/L^28, 47^, and HIF1α expression was detected via Western blot. The same approach was used to prepare HIF1α over-expression CD133^−^CD15^−^NESTIN^−^ glioma cells. To determine the influence of HIF1α intervening on neurosphere formation under hypoxic conditions, we performed sphere-formation assays as previously described. Moreover, we also determined the expression of related genes and proteins, including CD133, CD15, NESTIN, ABCG2 and MGMT, in 1%O_2_ following HIF1α interference with RT-PCR and Western blot. The influences on cell apoptosis and IC50 were determined following HIF1α intervening with flow cytometric and CCK-8.

### Preparation GL261-luc cells and implanted cells into C57 mice brain

The detail procedure of GL261-luc cells preparation was according to our last paper recently^31^. GL261-luc cells were then cultured in 1%O_2_ for 14d and induced the neurospheres formation. Digested neurospheres and implanted cells (3 × 10^4^ per each C57 mouse) into the brain of mice and fed them in 21%O_2_. The same treatment was done for control GL261-luc CD133^−^CD15^−^NESTIN^−^ cells. After feeding for 20d, we detected tumor volume by NightOWL Macro Imaging system (LB983 NC320, Berthold Technologies, Germany).

### Statistical analyses

For all statistical analyses, we used SPSS 19.0. The data are presented as the mean ± standard deviations (SDs). Statistical significance was set at *P* < 0.05, and all P values were two-sided. T test or one-way analysis of variance (one-way ANOVA) were used to evaluate differences when necessary.

## Electronic supplementary material

Supplementary Information
