# Supplementary material for: HIF1α regulates glioma chemosensitivity through the transformation between differentiation and dedifferentiation in various oxygen levels
Source: Sci Rep. 2017 Aug 11;7:7965. doi: 10.1038/s41598-017-06086-2 (PMC5554160; doi:10.1038/s41598-017-06086-2)

HIF1α regulates glioma chemosensitivity through the transformation between differentiation and dedifferentiation in various oxygen levels

Running title: HIF1α regulates glioma chemosensitivity

Pan Wang[1*], Wenwu Wan[1*], Shuanglong Xiong[2], Junwei Wang[1], Dewei Zou[1], Chuan Lan[1], Shuangjiang Yu[1], Bin Liao[1], Hua Feng[1], Nan Wu[1**]

[1]Department of Neurosurgery, Southwest Hospital, Third Military Medical University, Chongqing 400038, China

[2]Department of Oncology, Cancer Hospital, Chongqing 400030, China

*These authors contributed equally to this work.

**Correspondence should be addressed to: Dr. Nan. Wu, mailing address: Gaotanyan 30#, Shapingba, Department of Neurosurgery, Southwest Hospital, Third Military Medical University, Chongqing 400038, China. Tel and FAX: +86 23 68765265. E-mail: wunan881@tmmu.edu.cn

Supplementary Materials

Supplementary table legends

Table S1 Single CD133$^-$/CD15$^-$/NESTIN$^-$ glioma cell seeding and neurosphere formation rate detection More than 75% of cells survived after exposure to different oxygen levels for 3 d for both GL261 and U87 CD133$^-$CD15$^-$NESTIN$^-$ cells. In 1%O_2, neurospheres began to form after 1%O_2 exposure for 3 d. Approximately 20% of surviving GL261 and U87 cells (d7 spheres/d3 surviving cells) formed neurospheres after 7 d of exposure in the 1%O_2 microenvironment. The neurosphere rates then significantly increased, and 50.2%±4.167 of GL261 and 53.0%±3.391 of U87 (d14 spheres/d3 surviving cells) surviving cells formed neurospheres after hypoxia exposure for 14 d. More surprising, the sphere rates eventually reached 93.1%±5.541 from GL261 and 95.6%±2.665 from U87 (d21 spheres/d3 surviving cells) surviving cells under hypoxic conditions for 21 d. However, most cells remained as single cells and died after exposure to 21%O_2 or 95%O_2 for 21 d. In 21%O_2, only 1.60%±0.89 from GL261 and 1.10%±0.52 from U87 cells formed sparse and irregular aggregates, and no neurospheres were detected from cells grown in 95%O_2.

Table S2 IC50 and ratio detection among groups (A) The IC50 for GL261 CD133$^-$/CD15$^-$/NESTIN$^-$ cells after TMZ treatments increased 1.2-fold in 21%O_2 and 1.4-fold in 1%O_2 compared with the IC50 for cells under 95%O_2. The same results were obtained for U87 CD133$^-$/CD15$^-$/NESTIN$^-$ cells. The differences between 21%O_2 and 1%O_2 for GL261 and U87 CD133$^-$/CD15$^-$/NESTIN$^-$ cells were also significant, which demonstrated there was a higher IC50 in the 1%O_2 conditions. **(B)** The mean IC50 of GL261 CD133$^+$/CD15$^+$/NESTIN$^+$ neurospheres formed in 1%O_2 treated by TMZ was significantly higher, approximately 1.4-fold, than differentiated GL261 CD133$^-$/CD15$^-$/NESTIN$^-$ cells. The same results were obtained for U87 CD133$^+$/CD15$^+$/NESTIN$^+$ neurospheres formed in 1%O_2 treated by TMZ. **(C)** Fold increases in IC50 of 1.3- and 1.4-fold were observed in CD133$^+$/CD15$^+$/NESTIN$^+$ neurospheres in 1%O_2 compared with the cells in normoxia, and the IC50 was lower in 95%O_2 than in normoxia. **(D)** IC50 values decreased for HIF1α-silenced U87 CD133$^-$/CD15$^-$/NESTIN$^-$ cells after TMZ treatment under hypoxic conditions. **(E)** IC50 values increased for HIF1α-over-expressed U87 CD133$^-$/CD15$^-$/NESTIN$^-$ cells after TMZ treatment under hyperoxic conditions. **(F)** The IC50 decreased 1.4- and 2-fold for GL261 and U87 neurospheres, respectively, cultured with digoxin after TMZ treatments were identified compared with control cells without digoxin treatments.

Table S3 Single U87 CD133$^-$/CD15$^-$/NESTIN$^-$ HIF1α-ShRNA cell seeding and neurosphere formation rate detection in hypoxia conditions. HIF1α-silenced U87 CD133$^-$/CD15$^-$/NESTIN$^-$ cells presented a decreased neurosphere formation at 21 d. However, the

neurosphere formation rates were greater than 90% for both control and U87 vector cells, which were substantially higher than in HIF1α-silenced cells.

Table S4 Primary antibodies used for immunofluorescence and western blot.

Table S5 Primer sequences of related proteins in RT-qPCR.

Supplementary figure descriptions

Figure S1 Gray values of related proteins for $CD133^-CD15^-NESTIN^-$ cells cultured in different oxygen levels in western blot assays (A) GL261 $CD133^-CD15^-NESTIN^-$ cells presented significantly higher expression of MGMT and ABCG2 in 1%O_2 than in 95%O_2 for 24 or 48 h; increased expression was also identified in cells cultured in 21%O_2 compared with those cultured in 95%O_2 ($P<0.05$, One-way ANOVA). **(B)** A hypoxic environment promoted MGMT and ABCG2 expression in a time-dependent manner following exposure for 24 to 48 h ($P<0.05$, Paired-samples T Test). Under normoxic and hyperoxic conditions, the MGMT and ABCG2 levels remained stable without a significant difference between exposure for 24 and 48 h ($P>0.05$, Paired-samples T Test). **(C)** Decreased expression of CD133, CD15 and NESTIN after normoxia or hyperoxia exposure for 24 or 48 h for GL261 $CD133^-CD15^-NESTIN^-$ cells compared with cells cultivated in the hypoxic environment. There were significant differences between normoxia and hyperoxia. In contrast, the highest levels of GFAP were identified in 95%O_2, and the lowest expression levels were identified in 1%O_2 ($P<0.05$, One-way ANOVA). **(D)** A hypoxic environment promoted CD133, CD15 and NESTIN in a time-dependent manner from exposure for 24 to 48 h. In normoxia and hyperoxia, the expression of CD133, CD15 and NESTIN gradually decreased with statistical significance. In contrast, hypoxia inhibited the GFAP levels; normoxia and hyperoxia significantly promoted the expression of GFAP ($P<0.05$, Paired-samples T Test).

Figure S2 mRNA detection for neurospheres formed in 1%O_2. Increases of 3.3- to 5.7-fold of CD133, CD15, NESTIN, MGMT and ABCG2 expression were identified for GL261 neurospheres formed in 1%O_2 ($^*P<0.05$, Paired-samples T Test).

Figure S3 Gray values of related proteins for neurospheres cultured in different oxygen levels in western blot assays. (A) Cultured U87 cells which were digested from neurospheres in 1%O_2 or 21%O_2, and the results indicated there was reduced expression of CD133, CD15, NESTIN, MGMT and ABCG2 in cells exposed to 21%O_2 than in those exposed to 1%O_2. GFAP expression significantly increased after 21%O_2 exposure compared with that after 1%O_2 exposure ($^*P<0.05$, Paired-samples T Test). **(B)** Cultured U87 cells which were

digested from neurospheres in 21%O_2 or 95%O_2, and the results indicated there were lower expression levels of CD133, CD15, NESTIN, MGMT and ABCG2 in 95%O_2 than in 21%O_2 environments. The GFAP expression significantly increased after 95%O_2 exposure compared with 21%O_2 ($^{*}P<0.05$, $^{\#}P>0.05$, Paired-samples T Test). **(C-D)** CD133, CD15, NESTIN, MGMT and ABCG2 levels increased in a time-dependent manner in 1%O_2 exposure from 24 to 48 h. In contrast, normoxia and hyperoxia presented opposite effects, which resulted in an inhibition or stabilization of CD133, CD15, NESTIN, MGMT and ABCG2 expression. There was no GFAP detection in 1%O_2 and an increased expression of GFAP in a time-dependent manner in 21%O_2 and 95%O_2 exposure from 24 to 48 h ($^{*}P<0.05$, $^{\#}P>0.05$, Paired-samples T Test).

Figure S4 Gray values of HIF1α in western blot assays. (A) U87 $CD133^{+}/CD15^{+}/NESTIN^{+}$ cells digested from neurospheres cultured in 1%O_2 exhibited the highest levels of HIF1α, and the cells cultured in 95%O_2 presented the lowest expression of HIF1α ($^{*}P<0.05$, Paired-samples T Test). **(B)** Hypoxia maintained the HIF1α levels of U87 $CD133^{+}/CD15^{+}/NESTIN^{+}$ cells digested from neurospheres; in contrast, HIF1α expression decreased in a time-dependent manner from 21%O_2 exposure for 24 to 48 h. Under 95%O_2 conditions, HIF1α was not expressed ($^{*}P<0.05$, Paired-samples T Test).

Figure S5 Gray values of related proteins for HIF1α silencing cells in different oxygen levels. (A) Expression of HIF1α, CD133, CD15, NESTIN, MGMT and ABCG2 significantly decreased for U87 HIF1α silencing $CD133^{-}/CD15^{-}/NESTIN^{-}$ cells in a hypoxic environment; there were no differences between the U87 control and vector cells ($^{*}P>0.05$, $^{**}P<0.05$, One-way ANOVA). **(B)** Western blot assays indicated there were higher levels of HIF1α after over-expressing HIF1α for U87 $CD133^{-}/CD15^{-}/NESTIN^{-}$ cells in a hyperoxic environment ($^{*}P>0.05$, $^{**}P<0.05$, One-way ANOVA).

Figure S6 Neurosphere formation detection after HIF1α silencing under hypoxic conditions. (A) The neurosphere formation rate significantly decreased after HIF1α silencing under hypoxic conditions compared with the rate in U87 control and vector cells, and there were no difference in the neurosphere formation between U87 control and vector cells ($^{*}P<0.05$, $^{\#}P>0.05$, One-way ANOVA). **(B)** The size of neurospheres after HIF1α silencing under hypoxic conditions was substantially smaller than that for U87 control and vector cells. The mean cell number of each neurosphere (d21 cell number/neurosphere number) was approximately 1.7-fold higher for U87 control and vector cells than for the HIF1α-silenced group ($^{*}P<0.05$, $^{\#}P>0.05$, One-way ANOVA).

Figure S7 Negative control of immunofluorescence. A The negative control of IF for Figure 1B-C. **B** The negative control of IF for Figure 2A, D and Figure 6A. **C** The negative control of IF for Figure 3D. **D** The negative control of IF for Figure 4D and Figure 6E. **E** The negative control of IF for Figure 6D.

Figure S8 Neurospheres highly expressed GSCs markers. A RT-qPCR showed neurospheres formed in stem cells medium highly expressed CD133, CD15 and NESTIN (P<0.05, Paired-samples T Test). **B** WB showed comparing with normal glioma cells, neurospheres had higher expression of CD133, CD15 and NESTIN (P<0.05, Paired-samples T Test). C The rate of CD133, CD15 and NESTIN in GL261 $CD133^-CD15^-NESTIN^-$ cells was 9.65%, 0.545% and 7.40%, respectively. The rate of above was less than 10% in U87 $CD133^-CD15^-NESTIN^-$ cells. The rate of CD133, CD15 and NESTIN in GL261 $CD133^+CD15^+NESTIN^+$cells was 94.4%, 50.0% and 54.7%, respectively. The rate of CD133, CD15 and NESTIN in U87 $CD133^+CD15^+NESTIN^+$cells was 96.5%, 68.1% and 50.8%.

Figure S9 GL261-luc neurospheres formed by $CD133^-CD15^-NESTIN^-$ cells in hypoxia showed higher tumorigenic when implanting into C57 mice brain compared with $CD133^-CD15^-NESTIN^-$ cells cultured in normoxia with the same treatments ($^*P$<0.05, Paired-samples T Test) .

Supplementary table S1

Table S1 Single $CD133^-$/$CD15^-$/$NESTIN^-$ glioma cell seeding and neurospheres formation rate detecting

Cell/O_2	trials	0d seeding	3d		7d spheres (d7 spheres/d3 survived cells)	14d spheres (d14 spheres/d3 survived cells)	21d spheres (d21 spheres/d3 survived cells)
			survived cells	Spheres			
GL261 (1%O_2)	1	89	67	1	9(0.134)	32(0.478)	61(0.910)
	2	92	71	2	14(0.197)	38(0.535)	66(0.929)
	3	82	62	0	11(0.177)	29(0.468)	59(0.952)
	4	83	65	0	13(0.200)	37(0.569)	64(0.984)
	5	88	59	1	15(0.254)	28(0.475)	52(0.881)
	6	91	72	3	17(0.236)	35(0.486)	67(0.931)
	Mean	88	66	1.1	13(0.200)	33(0.502)	62(0.931)
	SE	4.135	5.060	1.170	2.858	4.167	5.541
GL261 (21%O_2)	1	84	71	0	0(0.000)	1(0.014)	1(0.014)
	2	86	62	1	1(0.016)	2(0.032)	2(0.032)
	3	91	73	0	0(0.000)	0(0.000)	0(0.000)
	4	85	64	1	1(0.016)	2(0.031)	2(0.031)
	5	81	62	0	1(0.016)	1(0.016)	1(0.016)
	6	86	65	0	0(0.000)	0(0.000)	0(0.000)
	Mean	86	66	0.3	0.5(0.008)	1(0.016)	1(0.016)
	SE	3.271	4.708	0.516	0.548	0.894	0.894
GL261 (95%O_2)	1	82	64	0	0(0.000)	0(0.000)	0(0.000)
	2	85	62	0	0(0.000)	0(0.000)	1(0.016)
	3	91	68	0	0(0.000)	0(0.000)	0(0.000)
	4	85	64	0	0(0.000)	1(0.016)	1(0.016)
	5	81	63	0	1(0.159)	2(0.032)	2(0.032)
	6	87	66	0	0(0.000)	0(0.000)	0(0.000)
	Mean	85	65	0	0.2(0.003)	0.5(0.008)	0.7(0.011)
	SE	3.601	2.168	0.000	0.408	0.837	0.816
U87 (1%O_2)	1	92	69	1	9(0.130)	33(0.478)	65(0.942)
	2	85	65	2	16(0.246)	36(0.554)	61(0.938)
	3	88	61	0	10(0.164)	30(0.492)	59(0.967)
	4	85	60	0	13(0.217)	36(0.600)	57(0.950)
	5	82	62	1	14(0.226)	29(0.468)	60(0.968)
	6	88	63	3	15(0.228)	37(0.583)	61(0.968)
	Mean	87	63	1.2	13(0.204)	34(0.530)	61(0.956)
	SE	3.445	3.266	1.169	2.787	3.391	2.665
U87 (21%O_2)	1	89	71	0	0(0.000)	0(0.000)	1(0.014)
	2	84	61	0	0(0.000)	1(0.016)	1(0.016)
	3	91	63	0	0(0.000)	0(0.000)	0(0.000)
	4	83	59	1	1(0.017)	1(0.017)	1(0.017)
	5	87	63	0	0(0.000)	0(0.000)	1(0.010)
	6	82	60	0	0(0.000)	1(0.017)	0(0.017)
	Mean	86	63	0.2	0.2(0.008)	0.5(0.008)	0.7(0.011)
	SE	3.578	4.309	0.408	0.408	0.009	0.516
U87 (95%O_2)	1	91	57	0	0(0.000)	0(0.000)	0(0.000)
	2	82	61	0	0(0.000)	0(0.000)	0(0.000)
	3	92	61	0	0(0.000)	0(0.000)	0(0.000)
	4	88	62	0	0(0.000)	0(0.000)	0(0.000)
	5	85	57	0	0(0.000)	1(0.018)	1(0.018)
	6	81	59	0	0(0.000)	0(0.000)	0(0.000)
	Mean	87	60	0	0(0.000)	0.2(0.003)	0.2(0.003)
	SE	4.593	2.172	0.000	0.000	0.408	0.408

Supplementary table S2

Table S2 IC50 and ratio detection among groups

A

Differentiation Cells	TMZ+O_2%	IC50 (μM)				
		Trials			Mean	Ratio
		1	2	3		
GL261	Hypoxia (1%)	533.261	531.069	653.584	572.638	1.4
	Normoxia (21%)	375.084	406.699	674.673	485.485	1.2
	Hyperoxia (95%)	198.257	392.156	645.732	412.048	1.0
U87	Hypoxia (1%)	524.102	1218.439	829.499	857.346	1.2
	Normoxia (21%)	517.383	1127.783	817.463	820.876	1.1
	Hyperoxia (95%)	460.591	1049.945	672.979	727.838	1.0

B

Neurospheres Cells formed in 1%O_2	Normoxia 21%O_2	IC50 (μM)				
		Trials			Mean	Ratio
	TMZ+Neurospheres	1	2	3		
GL261	$CD133^{-}CD15^{-}$ $NESTIN^{-}$ Cells	805.217	1051.438	1064.454	973.703	1.0
	Neurospheres Cells	1260.495	1580.223	1355.977	1298.898	1.4
U87	$CD133^{-}CD15^{-}$ $NESTIN^{-}$ Cells	608.031	517.383	742.300	622.5713	1.0
	Neurospheres Cells	1741.341	1093.940	1112.070	1315.783	2.1

C

Stem Cells	TMZ+O_2%	IC50 (μM)				
		Trials			Mean	Ratio
		1	2	3		
GL261	Hypoxia (1%)	1499.990	1242.14	1531.621	1424.585	1.4
	Normoxia (21%)	804.666	974.068	1361.857	1046.864	1.0
U87	Hypoxia (1%)	784.281	880.274	1262.796	975.7839	1.3
	Normoxia (21%)	515.115	702.496	1104.661	774.0907	1.0

Stem Cells	TMZ+O_2%	IC50 (μM)				
		Trials			Mean	Ratio
		1	2	3		
GL261	Normoxia (21%)	1112.43	1313.899	1439.905	1288.746	1.4
	Hyperoxia (95%)	903.603	761.419	1092.236	919.086	1.0
U87	Normoxia (21%)	942.3	1193.943	1841.34	1325.861	1.2
	Hyperoxia (95%)	871.438	929.14	1570.08	1123.552	1.0

D

Differentiation Cells	Hypoxia 1%O_2	IC50 (μM)				
		Trials			Mean	Ratio
	TMZ+ HIF1α	1	2	3		
U87	Con	622.113	705.549	634.52	654.0603	1.0
	Vector	682.75	643.884	643.79	656.809	1.0
	HIF1α-ShRNA	392.156	457.148	521.89	457.0647	0.7

E

Differentiation Cells	Hyperoxia 95%O_2	IC50 (μM)				
		Trials			Mean	Ratio
	TMZ+ HIF1α	1	2	3		
U87	Con	1162.796	1216.576	1280.224	1219.865	1.0
	Vector	1190.499	1232.654	1296.37	1239.841	1.0
	HIF1α-overexpression	1430.468	1445.952	1489.442	1455.287	1.2

F

Stem cells	Hypoxia 1%O_2	IC50 (μM)				
		Trials			Mean	Ratio
	TMZ+digoxin	1	2	3		
GL261	-digoxin	2047.336	1843.423	1708.264	1866.341	1.0
	+digoxin	1067.219	1487.199	1636.371	1396.930	0.7
U87	-digoxin	1279.857	880.274	1090.537	1083.556	1.0
	+digoxin	474.684	633.55	675.581	594.605	0.5

Supplementary table S3

Table S3 Single U87 CD133$^-$/CD15$^-$/NESTIN$^-$ HIF1α-ShRNA cell seeding and neurospheres formation rate detecting

Cell	trials	0d seeding	3d		7d spheres (d7 spheres/d3 survived cells)	14d spheres (d14 spheres/d3 survived cells)	21d spheres (d21 spheres/d3 survived cells)
			survived cells	Spheres			
U87 HIF1α not targeted	1	92	62	2	26 (0.419)	37(0.597)	59(0.952)
	2	88	60	3	24(0.400)	42(0.700)	60(1.000)
	3	91	59	1	25(0.424)	38(0.644)	58(0.983)
	4	93	65	3	22(0.338)	46(0.708)	62(0.954)
	5	87	58	0	21(0.362)	42(0.724)	55(0.948)
	6	88	59	1	27(0.457)	39(0.661)	56(0.949)
	Mean	90	61	1.7	24(0.400)	41(0.672)	58(0.964)
	SE	2.483	2.588	1.211	2.317	3.326	2.582
U87 HIF1α Vector	1	95	63	1	28(0.444)	39(0.619)	56(0.889)
	2	90	59	3	22(0.372)	39(0.661)	58(0.983)
	3	91	57	2	25(0.439)	35(0.614)	54(0.947)
	4	92	61	2	23(0.377)	44(0.721)	56(0.918)
	5	92	60	3	21(0.350)	45(0.750)	57(0.950)
	6	89	58	0	25(0.431)	37(0.628)	54(0.931)
	Mean	92	60	1.8	24(0.400)	40(0.667)	56(0.936)
	SE	2.074	2.160	1.169	2.530	3.920	1.602
U87 HIF1α ShRNA	1	92	59	0	11(0.186)	28(0.475)	41(0.695)
	2	94	57	1	13(0.228)	32(0.561)	38(0.667)
	3	93	61	1	16(0.262)	28(0.459)	36(0.590)
	4	92	59	2	12(0.203)	32(0.542)	39(0.661)
	5	88	62	0	17(0.274)	33(0.532)	41(0.661)
	6	91	63	0	13(0.206)	41(0.651)	45(0.714)
	Mean	92	60	0.7	14(0.226)	32(0.537)	40(0.665)
	SE	2.066	2.229	0.816	2.338	4.761	3.098

Supplementary table S4

Table S4 Related information on primary antibodies of immunofluorescence and western-blot

Antibodies	ID (Human)	ID (Mouse)	Company and country	Immunofluorescence (Ration)	Western-blot (Ration)
CD133	MBS462020	MBS462020	MyBiosource USA	1:150	1:1000
CD15	MAB2155	MAB2155	R&D Systems USA	1:100	1:1000
NESTIN	MAB1259	MAB2736	R&D Systems USA	1:100	1:1000
GFAP	MAB2594	MAB2594	R&D Systems USA	1:100	1:1000
ABCG2	ab130244	ab130244	Abcam USA	1:100	1:1000
MGMT	AF3794	AF3299	R&D Systems USA	1:100	1:1000
HIF1α	MAB1536	MAB1536	R&D Systems USA	1:100	1:1000

Supplementary table S5

Table S5 the primer sequences of related proteins

	upstream (5'→3')	downstream (5'→3')
Human CD133	GCCCCCAGGAAATTTGAGGAAC	GCTTTGGTATAGAGTGCTCAGTGATTG
Human CD15	TGGGCAGGCTGGTCTTGAACT	CACGGCGGCTCACACCTGTA
Human NESTIN	GCCCCTGGTGGAAGATGATG	GCCCTGAACCCTCTTTGCCTC
Human GFAP	GCTGCTAGAGGGCGAGGAGA	CGGCGGCGTTCCATTTAC
Human ABCG2	TAGTGAGGAAAGTTCCTGTC	AGCTCAGTTAACTCCTGTAAG
Human MGMT	TCCCGTTTTCCAGCAAGAGTC	GGATGAGGATGGGGACAGGATT
Human HIF1α	CCCTCACCCACAAAAATTAC	GGGACTATTAGGCTCAGGTGAAC
Mouse CD133	CCGCGATGGACTCTGCTGTTAATG	GGGCACAGTCTCAACATCGTCGTATAC
Mouse CD15	ATCGGGCTGCTGCACACTG	AGCGGAAGTAGCGGCGATAGAC
Mouse NESTIN	GCCCAAGCAGGTGAACAAGACT	CAGCCCTTGCATTCCAGAGTCT
Mouse GFAP	GGCGAGCGAGCGTGCAGAGA	CCCGAAGCTCCGCCTGGTAGAC
Mouse ABCG2	AGAAACTCTTCATACATGAGTACA	AAGTGTTGCTACAGACACCACAC
Mouse MGMT	GCCAGAGGGAGTTCCAGAGCC	CCGCTTTGGGGTTGCCTGCC
Mouse HIF1α	AGCCTTAACCTGTCTGCCACTTTG	GGGCACAGTCTCAACATCGTCGTATAC
β-actin	ACCCGCCGCCAGCTCACC	GGGGGGCACGAAGGCTCATC

Supplementary Figure S1

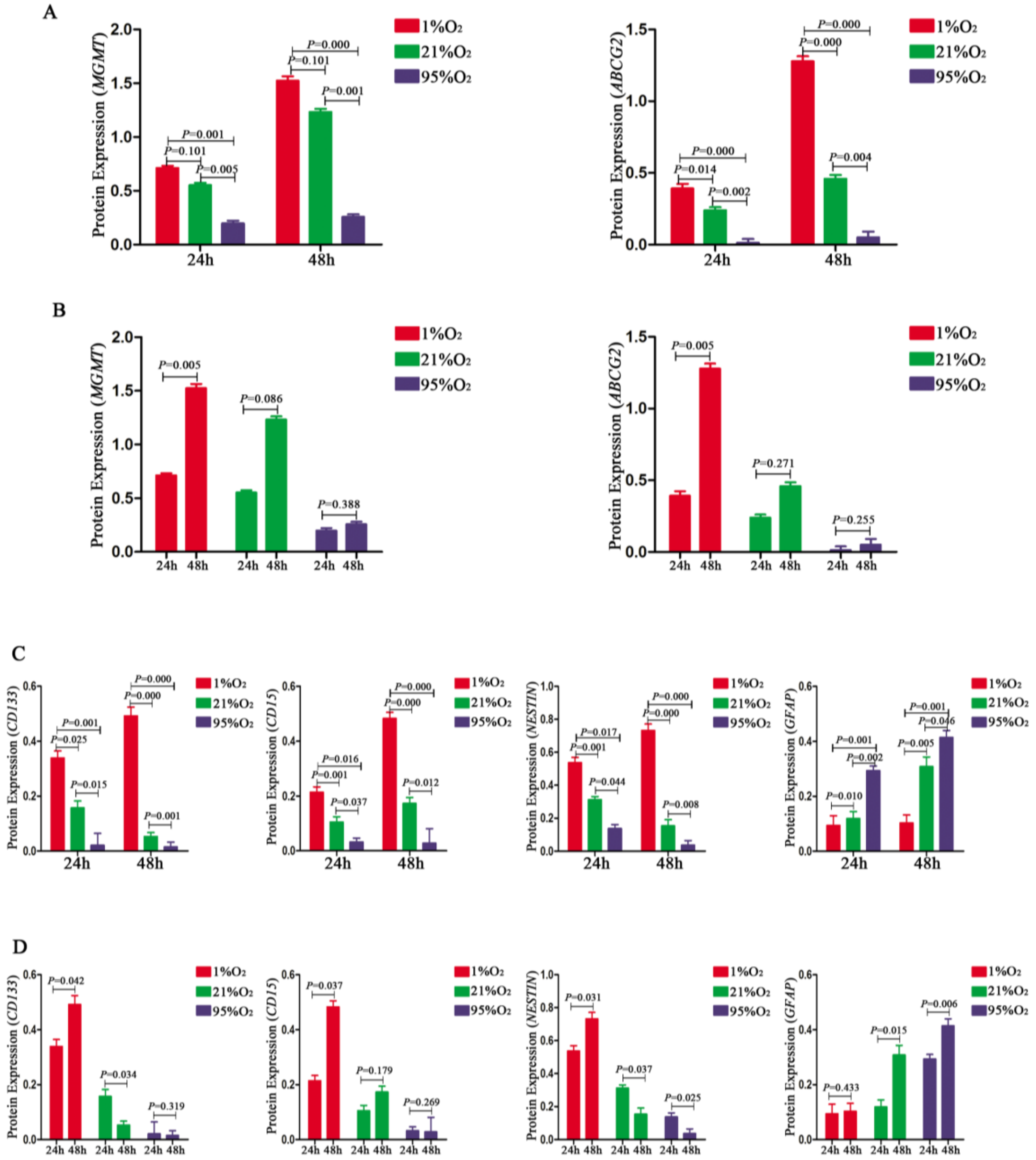

Supplementary Figure S2

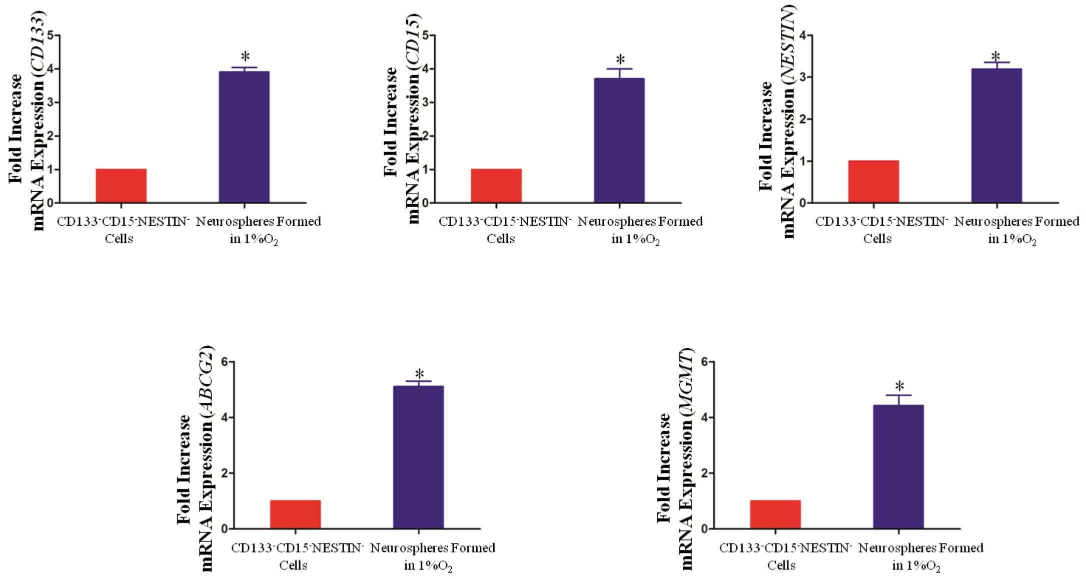

Supplementary Figure S3

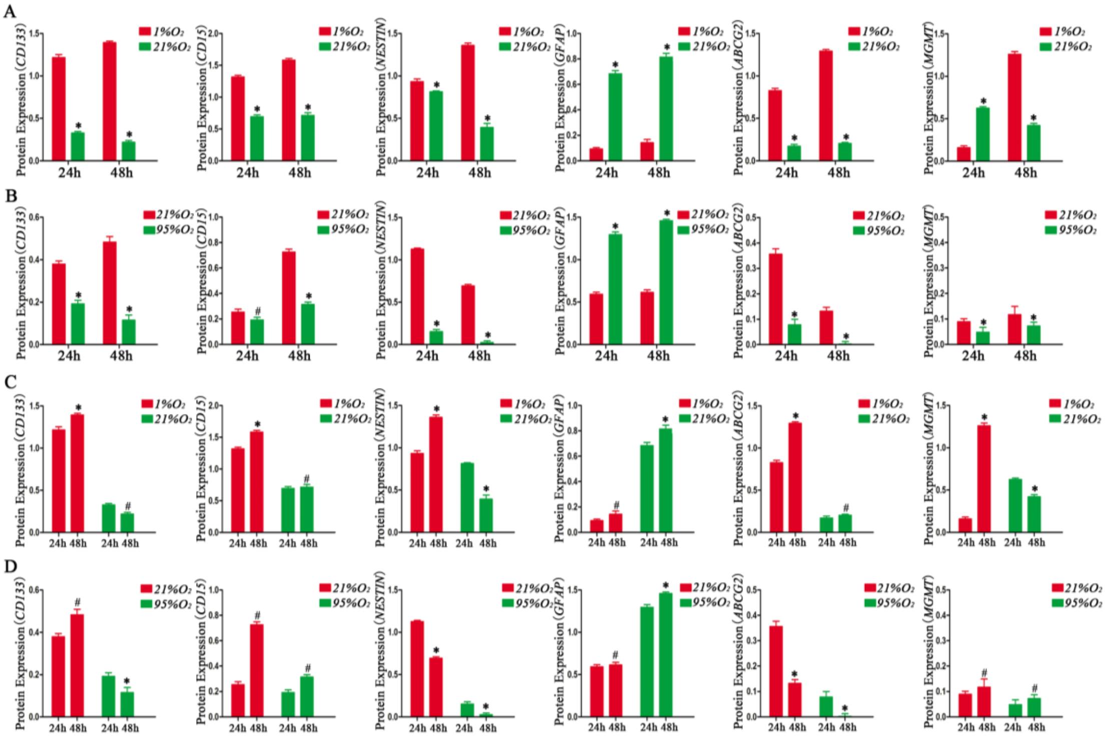

Supplementary Figure S4

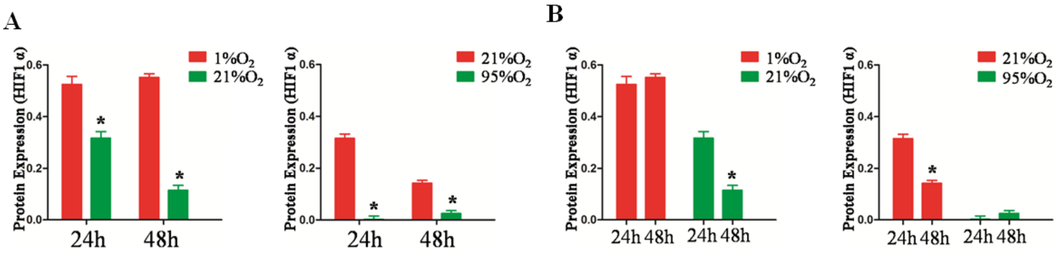

Supplementary Figure S5

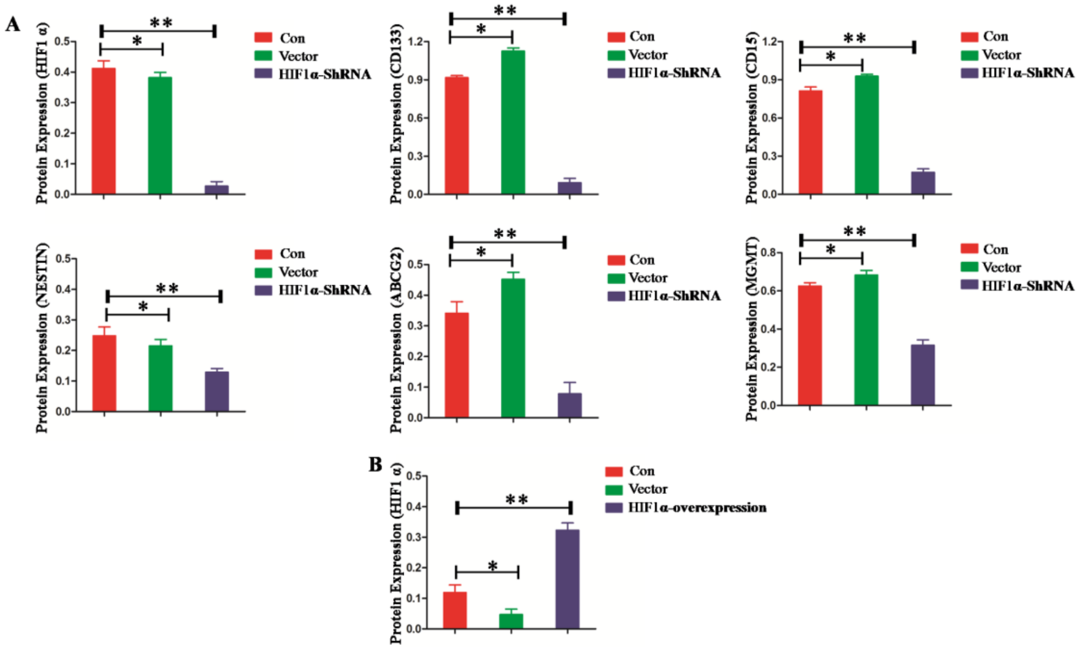

Supplementary Figure S6

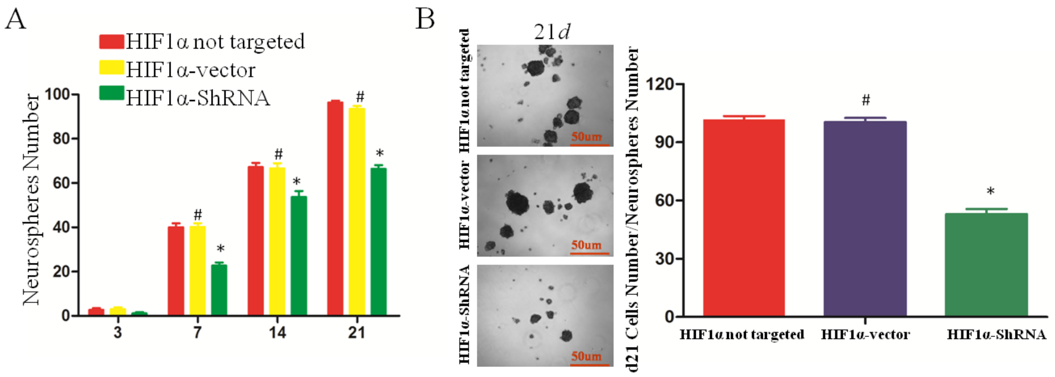

Supplementary Figure S7

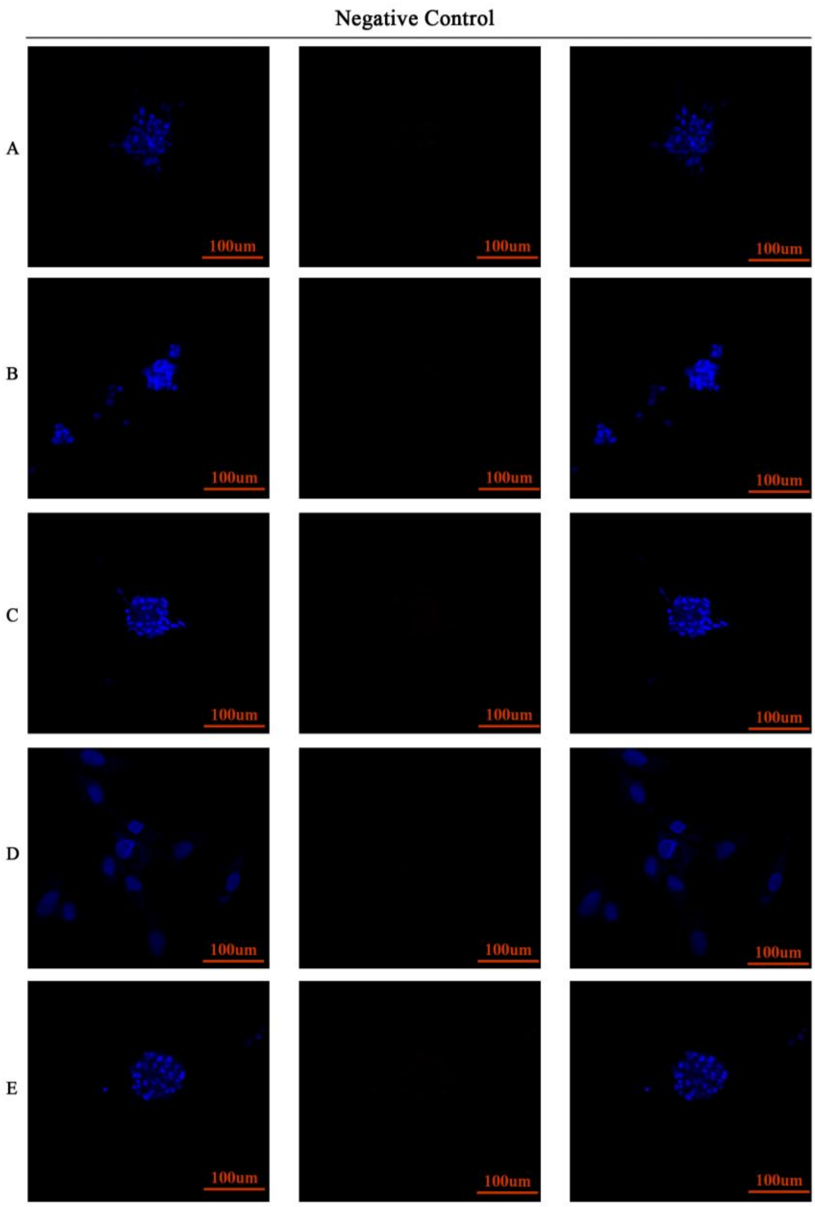

Supplementary Figure S8

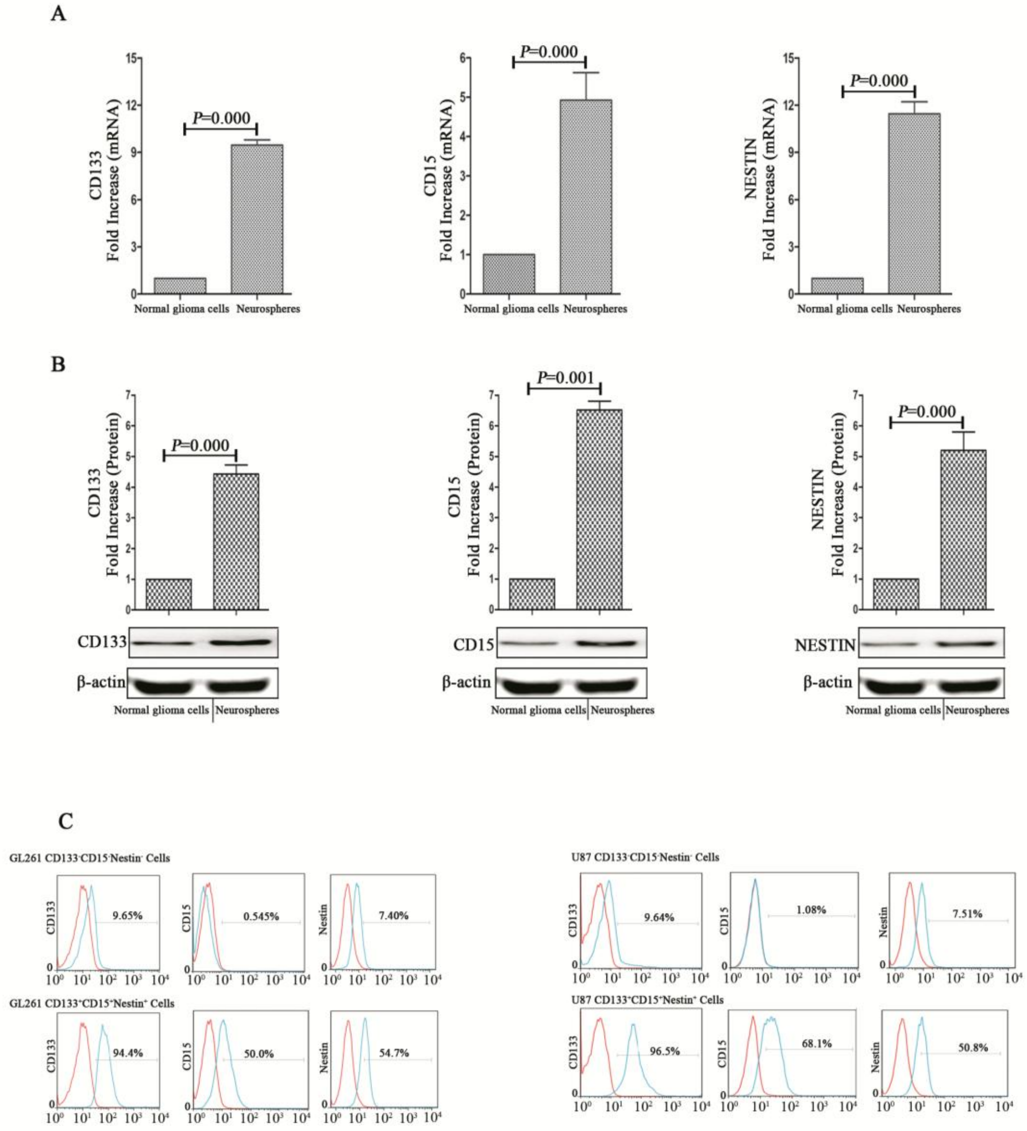

Supplementary Figure S9

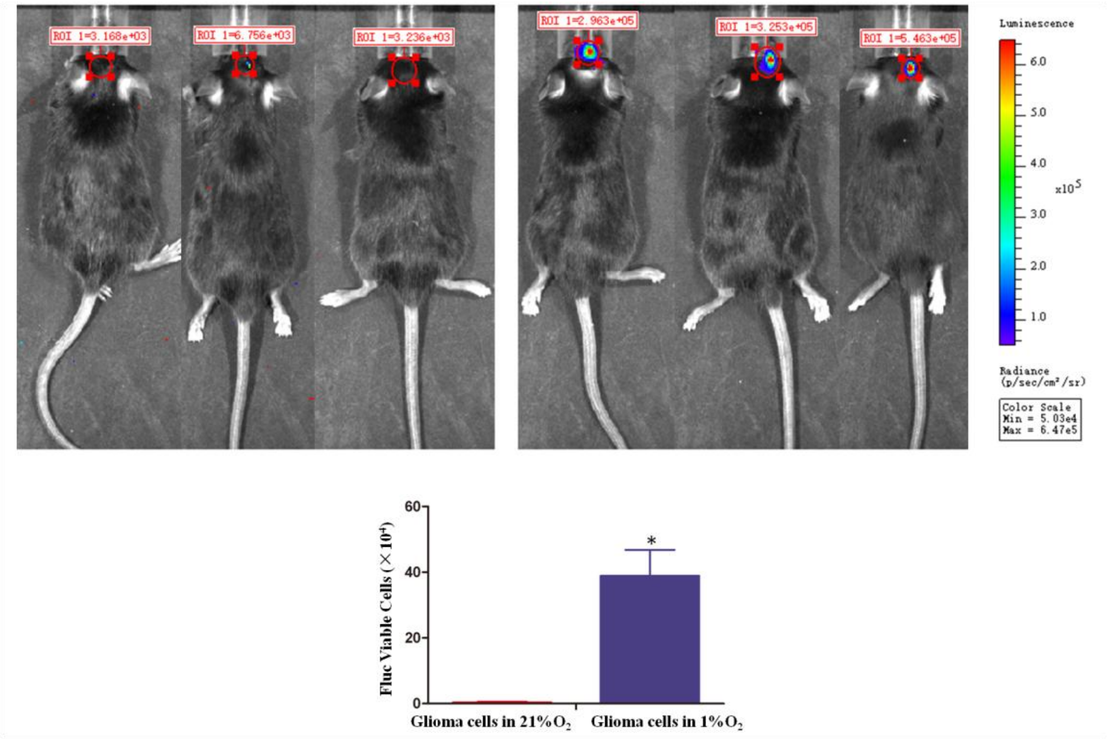

Supplement: Supplementary file 1 — Supplementary Information [file 41598_2017_6086_MOESM1_ESM.pdf]
